# ECIRA - European crop-specific irrigated area at 1 km resolution annually from 2010 to 2020

**DOI:** 10.1038/s41597-025-05628-y

**Published:** 2025-08-04

**Authors:** Wanxue Zhu, Josef Baumert, Hugo Storm, Thomas Heckelei, Stefan Siebert

**Affiliations:** 1 Department of Crop Sciences, University of Göttingen, Von-Siebold-Str. 8, 37075 Göttingen, Germany; 2 Institute for Food and Resource Economics (ILR), University of Bonn, Bonn, Germany

**Keywords:** Hydrology, Agriculture, Water resources

## Abstract

Irrigation significantly contributes to total water withdrawal and exhibits considerable spatial and temporal variability, particularly in more humid regions. This variability is caused by climate, soil properties, and crop water requirements. However, time series of high-resolution, crop-specific irrigated area data remain scarce in Europe. We developed and applied a method to harmonize input data on crop types and irrigation to obtain the European Crop-specific IRrigated Area (ECIRA) dataset, providing annual 1-km gridded crop-specific irrigated area for 16 crop types across 28 European countries for 2010–2020. The ECIRA dataset effectively identifies crop-specific irrigated hotspots, aligns with subnational census data, and strongly agrees with LUCAS field observations and other survey-based crop-specific irrigation area datasets. However, caution is needed for region- and location-specific studies, as the Europe-wide scope of ECIRA entails a trade-off between local details and overall consistency. It can be used in assessments of crop productivity and crop water use, as input in land surface-, crop-, and hydrological modeling, in climate impact studies and to support improved water resources management.

## Background & Summary

Irrigation is the largest component of human freshwater demand and significantly impacts the global hydrological cycle^1^. Despite accounting for just 20% of the worldwide cultivated land, irrigated agriculture contributes to 40% of global crop production^2^. In Europe, irrigation practices exhibit notable regional disparities due to diverse environmental, socio-economic, and hydrological factors^3^. Irrigation is indispensable for agricultural production in Mediterranean regions, whereas in Central and Northern Europe, it primarily supplements precipitation particularly during critical growth stages in dry years^3,4^. Irrigation is also proposed as a measure to adapt to climate change; however, climate change threatens to modify water and energy cycles through rising temperatures and altered precipitation patterns, exacerbating water scarcity, especially in already water-stressed regions^5^. These climate alterations, combined with population growth and inter-country competition, are anticipated to increase water withdrawals, potentially undermining other developmental and environmental goals^6,7^.

Irrigation water usage is influenced by various factors, including climate, soil properties, crop type and growing season, irrigation efficiency, and socio-economic aspects^5,8^. Process-based models incorporate these factors into ecological, hydrological, and physiological processes to estimate irrigation water requirements^9,10^. Such modeling studies require data on irrigated area, crop evapotranspiration equations, and site-specific irrigation efficiency parameters^11^. Among these factors, the irrigated area has been identified as a key driver of irrigation water withdrawals^12,13^. In Europe, the variability of irrigated area is particularly pronounced in temperate zones; for example, the total irrigated area in the Netherlands was 62 thousand hectares in 2003, while it reached 201 thousand hectares in 2016^14^. Such remarkable variations underscore the necessity to account for inter-annual variability in irrigated area to enhance the accuracy of process-based model simulations^15^, which in turn improves understanding of water balances and facilitates better scenario projections as well as agricultural management^16^.

In recent decades, remote sensing has emerged as an appropriate approach for large-scale ground surface monitoring^17,18^. It has proven to be highly effective for mapping irrigation area in semiarid and arid regions at high resolution^19^, supported by several reasonable assumptions. These include the dependency of crops on irrigation in extremely dry areas^20^, the distinct spectral, spatial, and temporal characteristics of irrigated crops compared to rainfed crops^21–23^, and water balances between evapotranspiration and the sum of precipitation and irrigation^17,19^. However, the remote sensing approach encounters considerable challenges in mapping irrigation area in humid regions such as Western and Northern Europe, due to data gaps caused by cloud coverage, weak irrigation signals, and time lags between satellite observations and application of irrigation^3,24,25^. In contrast, farm surveys, which typically employ questionnaires about irrigation practices throughout the year address some of the limitations of remote sensing^26^. Consequently, farm surveys may be particularly relevant for irrigation mapping of a smaller extent in humid regions where irrigation exhibits high interannual temporal and spatial variability.

Several datasets provide information on the spatial distribution and extent of irrigation areas globally or specifically within Europe (Table 1). Among these, the Global Irrigation Area Map (GIAM)^23^, Global Rainfed, Irrigated, and Paddy Cropland Extent (GRIPC)^27^, Z-2019^28^, Global Maximum Irrigation Extent (GMIE)^29^, Global 30 m Land-cover Dynamics Monitoring Dataset (GLC_FCS30D)^30^, and CORINE Land Cover datasets^31^ were primarily developed using remote sensing data, while others were mainly based on statistical survey data. Typically, remote sensing-based products offer higher spatial resolution (e.g., GMIE at 30 m and GRIPC at 500 m) compared to those based on statistical survey data (e.g., the Global Map of Irrigation Area – GMIA^15,32^ – and Spatial Production Allocation Model dataset – SPAM^33^ – at 5 arc minutes resolution, corresponding to approximately 9.3 km at the equator), mostly owing to the characteristics of the original data sources. It should be noted that the definition of irrigation area varies across datasets. For example, the GMIE dataset provides the maximum irrigation area for the periods 2010–2019 and 2017–2019^29^, but it cannot be directly equated to either irrigable or irrigated area. Additionally, some datasets such as the SPAM and GLC_FCS30D primarily focus on land cover and land use classification rather than specifically distinguishing irrigation (Table 1). This focus can introduce large uncertainties in detecting variations in irrigation areas^34^.

**Table 1:** Summary of the main global datasets on irrigation area since the 1950s.

Data source	Coverage	Datasets	Irrigation information	Reference year (temporal resolution)	Spatial resolution
Survey	Global	*FAOSTAT^37^	AAI & AEI	From 1961 (yearly)	Country
		*GMIA^15,32^	AEI	Around 2000, 2005	5 arc min
		*HID^35,51^	AEI	1900–1980 (10 yearly), 1985–2015 (5 yearly)	5 arc min
		*MIRCA 2000^38^	AAI (crop)	Around 2000 (monthly)	5 arc min
		*MIRCA-OS^39,40^	AAI (crop)	2000, 2005, 2010, 2015 (monthly)	5 arc min
		*M18^36^	AEI	Around 2005	30 arc sec
		SPAM^33^	AEI (crop)	2000, 2005, 2010, 2017	5 arc min
		GAEZ^34^	AAI (crop)	2015	5 arc min
RS	Global	*GIAM^23^	AAI (source)	Around 2000	10 km
		*GRIPC^27^	AAI (rice)	Around 2005	500 m
		*Z-2019^28^	AAI	2015	25 km
		*GMIE^29^	Max AAI	2010–2019, 2017–2019 periods	30 m
		GLC_FCS30D^30^	AAI	1985–2000 (5 yearly), 2000–2022 (yearly)	30 m
Survey	Europe	*Eurostat FSS^14,41,43^	AAI & AEI (crop, type, source)	1990–2020 (2–3 yearly)	Mainly NUTS2
		*ELIAD	AAI & AEI	1990–2020 (yearly)	Mainly NUTS2
		*EIM 2000^4^	AAI (crop)	Around 2000	100 m
		*EIM 2010^3^	AAI (crop)	Around 2010	10 km
		*LUCAS^42^	AAI & AEI (crop, type, source)	2009, 2012, 2015, 2018	Point level
RS	Europe	CORINE Land Cover^31^	Non-irrigated arable land & permanently AAI	2000–2018 (6 yearly)	100 m

Note: **Main data source**: *RS* denotes datasets primarily generated using remote sensing data, while *Survey* signifies datasets generated mainly using statistical survey data. **Datasets**: Datasets with * denote that they are specifically focused on irrigation. GMIA is the global map of irrigation area dataset; HID is the global historical irrigation dataset; MIRCA 2000 is the global monthly irrigated and rainfed crop areas around 2000; MIRCA-OS is open-source MIRCA; SPAM is spatial production allocation model dataset; GAEZ is Global Agro-Ecological Zones dataset; GIAM is the global irrigated area map; GRIPC is global rainfed, irrigated and paddy croplands dataset; GMIE is global maximum irrigation extent; GLC_FCS30D is global 30 m land-cover dynamics monitoring dataset; FSS is farm structure survey; ELIAD is European long-term irrigation area dataset; EIM is European irrigation map; LUCAS is land use/cover area frame statistical survey. **Irrigation information**: ‘AAI’ is the area actually irrigated while the ‘AEI’ is the area equipped for irrigation; ‘type’ means information of different irrigation types is included; ‘crop’ means crop-specific irrigation information is included; ‘source’ means the information of water source for irrigation is provided; ‘rice’ means the irrigation information of rice is separately generated.

Specifically, in terms of temporal resolution (Table 1), most irrigation datasets focus on specific reference years such as 2000, 2005, 2010, and 2015, which limits their ability to detect interannual dynamics in irrigation^3,15,35,36^. Although FAOSTAT has provided global annual irrigation area data since 1961, this data is at the national level and often involves gap-filling through simple interpolation or estimates, leading to large uncertainty, particularly in regions with high irrigation variability^37^. The European Long-term Irrigation Area Dataset (ELIAD)^26^ was developed specifically for Europe, providing an annual time series of total irrigated and irrigable area across 32 European countries from 1990 to 2020. ELIAD is provided for NUTS2 regions (except for the UK and Germany are at the NUTS1 level; NUTS is Nomenclature of Territorial Units for Statistics) and does not differentiate between crop types. The GLC_FCS30D dataset provides annual land cover data from 2000 to 2022^30^, but its irrigation information for Europe may not be particularly reliable. This is because GLC_FCS30D prioritizes land cover classification over detecting irrigation signals, with its primary data source being the Landsat satellite series images, which are sensitive to cloud cover.

In terms of distinguishing crop types (Table 1), the Global Monthly Irrigated and Rainfed Crop Areas around 2000 (MIRCA 2000)^38^, MIRCA open source (MIRCA-OS)^39,40^, SPAM^33^, Eurostat Farm Structure Survey^41^, European Irrigation Map (EIM) 2000^4^, EIM 2010^3^, and Land Use/Cover Area Frame Statistical Survey (LUCAS)^42^ datasets provide crop-specific irrigation area information. It is noteworthy that these datasets primarily rely on statistical survey data rather than remote sensing observations. Consequently, except for EIM2000 with a 100-meter resolution, other crop-specific irrigation area datasets have coarser resolutions like 10 km and 5 arc minutes (Table 1). Additionally, regarding both temporal resolution and crop type identification, MIRCA 2000, EIM 2000, and EIM 2010 are only available for the fixed reference years of 2000 or 2010. SPAM is updated approximately every 5 years, but its irrigation information is derived from GMIA v5.0 irrigable area data with a fixed reference year. MIRCA-OS^39,40^ provides global crop-specific irrigated area monthly for the years 2000 to 2015, with updates every five years; its irrigation data for Europe are sourced from Eurostat. Eurostat Farm Structure Survey updates total and crop-specific irrigable and irrigated data every 2–3 years but only at the subnational level (mainly at NUTS2)^14,41,43^, with large temporal data gaps, particularly in western and northern Europe. LUCAS data is updated every three years since 2009 (with no irrigation information collected in the LUCAS 2006 survey) but collects information at the point level with only a single field observation, which may not sufficiently capture a regional overview and may overlook irrigation practices occurring at other times. Therefore, there is currently no dataset for Europe that provides both time-series annual updates and crop-specific irrigation area information.

To address the irrigation area data gap, this study developed the annual European Crop-specific IRrigated Area dataset (ECIRA) from 2010 to 2020, featuring 16 crop types at a 1 km spatial resolution. The ECIRA dataset is suitable for European-scale modeling applications due to its fine temporal and spatial resolutions, enhancing scenario development at various spatial scales as well as better serving water resources management.

## Methods

In this study, the definition of irrigable and irrigated area, and utilized agricultural area aligns with those in Eurostat^44^, as the main data source – ELIAD^26,45^ and the Probabilistic Data Generating Process-based Crop Type Map (DGPCM)^46,47^ – were primarily generated using the Eurostat Farm Structure data. Area equipped with irrigation infrastructure (AEI, also known as irrigable area, in hectares) is defined as the maximum area that could be irrigated in a year using available equipment and water. Area actually irrigated (AAI, also known as irrigated area, in hectares) refers to the total area of crops that have been irrigated at least once over the preceding 12 months, excluding areas occupied by greenhouses and kitchen gardens. If more than one crop is grown in a field during the harvest year, the area should only be indicated once: for the main crop, if irrigation was used for it, or otherwise for the most important irrigated secondary or successive crop. Note that it can happen that an area is equipped for irrigation but irrigation is not necessary or not possible in a given year. Hence, AEI and AAI can deviate and they measure two different aspects, and AAI should be smaller than or equal to AEI. Irrigation area can refer to either AAI or AEI when distinguishing the actual irrigation is not crucial.

The total irrigation area refers to the aggregated irrigation area without distinguishing between crop types. The percentage of AAI relative to AEI (AAI/AEI, %) is referred to as the irrigation percentage. AEI percentage is the proportion of irrigable area to the total grid area within each grid (%); AAI percentage is defined analogously. Utilized agricultural area (UAA) encompasses all land used for agricultural purposes by the holding, including arable land, permanent grassland, permanent crops, and kitchen gardens, regardless of tenure type or if it is used as a part of common land^48^. The reference year signifies that the data encompasses information from around that time, potentially incorporating data from the years before and after it, rather than being confined strictly to that single year, due to factors such as data availability.

### Main input datasets

**Fig. 1:**
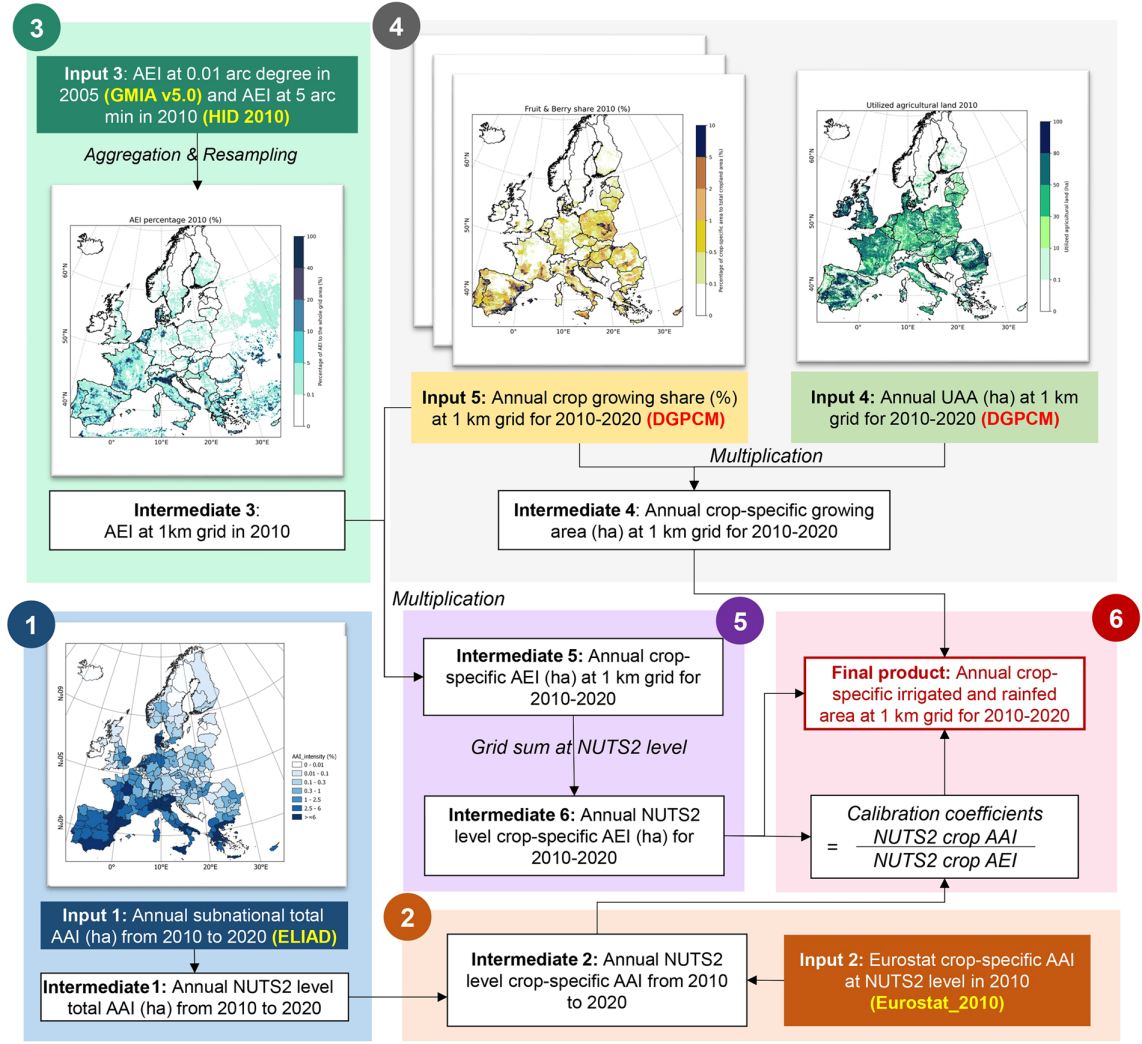
Flowchart of generating European Crop-specific IRrigated Area (ECIRA) dataset at 1 km gridded resolution annually for the year 2010–2020. AEI is the cropland area equipped with irrigation infrastructure; AAI is the cropland area that actually receives irrigation at least once per year. ELIAD is the European long-term irrigation area dataset^26^. DGPCM is the Probabilistic Data Generating Process-based Crop Type Map^46^. GMIA is the Global Map of Irrigation Area dataset^15,32^. HID is the global Historical Irrigation Dataset^35,51^. NUTS is the Nomenclature of Territorial Units for Statistics. The number in the upper left corner of the box indicates the step order for ECIRA dataset generation.

The primary input data for generating ECIRA encompass annual total AAI mainly at the NUTS2 level from 2010 to 2020 (sourced from ELIAD^26,45^), NUTS2 level crop-specific AAI in 2010 (sourced from Eurostat_2010^41^), total AEI in 2005 at 0.6 arc minute (corresponding to 1.11 km at the equator, sourced from GMIA v5.0^32,49^) and total AEI in 2010 at 5 arc minute (sourced from Historical Irrigation Dataset - HID^35,50^), as well as the 1-km gridded annual UAA and crop growing share for 2010–2020 (sourced from DGPCM^46,47^) (Fig. 1 and Table 2). Considering data availability, 28 European countries (the 27 current EU member states and the United Kingdom) are selected in this study (Table S1).

**Table 2:** Summary of datasets used to develop European crop-specific irrigated area dataset (ECIRA) from 2010 to 2020.

Abbreviations	Full name	Information provided	Temporal resolution	Spatial resolution
ELIAD^26,45^	European Long-term Irrigation Area Dataset	Total irrigated area ($\widehat{TAAI}$)	Yearly	NUTS1 or NUTS2
Eurostat_2010^41^	Reported data by EUROSTAT in the year 2010	Crop-specific irrigated area (*AAI*_*c*_)	Fixed in 2010	NUTS2
		The sum of crop-specific irrigated area (*TAAI*)		
GMIA v5.0^32^	The Global Map of Irrigation Area version 5.0	Total irrigable area (*TAEI*_*0.6min, Y2005*_)	Fixed in 2005	0.6 arc min
HID^35,51^	Historical Irrigation Dataset	Total irrigable area (*TAEI*_*5min*_, _*Y2010*_)	Fixed in 2010	5 arc min
DGPCM^46,47^	Probabilistic Data Generating Process-based Crop Type Map	Utilized agricultural area (UAA) & Crop growing share (CGS)	Yearly	1 km grid

The ELIAD dataset provides annual subnational data on total irrigation area for 32 European countries from 1990 to 2020^26^ (Fig. 1 and Table 2). ELIAD is primarily derived from Eurostat, FAOSTAT, and national statistical surveys, with corrections made to the irrigation reference year. Its development considered the availability of irrigation infrastructure and the impacts of wetness and drought on the use of irrigation infrastructure. The total AAI is provided at the NUTS2 level, except for the UK and Germany, where it is only available at the NUTS1 level due to data limitations.

The Eurostat_2010 dataset provides total and crop-specific AAI at the NUTS2 level across 28 European countries for 2010^41^ (Fig. 1 and Table 2), collected from the EU Agriculture Census. However, the sum of crop-specific AAI does not always match the reported total AAI in some NUTS2 units and this discrepancy is challenging to address due to the lack of additional ancillary information. Therefore, the proportion of AAI for each crop relative to the sum of crop-specific AAI was calculated, which was then employed to disaggregate the annual total AAI from ELIAD into crop-specific AAI for 2010–2020.

The GMIA v5.0^32^ dataset provides global AEI around the year 2005 at a 5-arc minute resolution, while the European regions have a higher resolution of 0.6 arc minute due to the enhanced data availability (Fig. 1 and Table 2). The HID^35,51^ dataset provides global AEI at a 5-arc minute level from 1900 to 2015 and is updated every 10 or 5 years. Both HID and GMIA v5.0 AEI data were generated mainly from national statistical surveys, and they also integrated information from reports, yearbooks, and land cover maps. These two datasets maintain a high degree of consistency between the total irrigation area of subnational grid cells and statistical data by applying various conditional constraints^15^. Since no AEI data is available for 2020 and crop-specific AAI is only available in 2010 (in Eurostat_2010) during the 2010–2020 period, we utilized a consistent AEI percentage temporally fixed in 2010 (HID 2010). However, as the AEI in 2005 from the GMIA v5.0 has a higher spatial resolution, we combined the HID (5 arc minutes, for the year 2010) and GMIA v5.0 (0.6 arc minutes, for the year 2005) to generate the AEI 2010 at the 1 km gridded level.

The DGPCM dataset provides ‘cell weights’ (proportional to the UAA in a grid cell) and crop cultivation shares for 28 crop types across 28 European countries at the 1 km level for 2010–2020^46,47^ (Fig. 1 and Table 2). It is based on a probabilistic model of the data-generating process that uses climate, soil, and topography data to estimate crop probabilities at the cell level and links them with subnational administrative crop acreage information. DGPCM is therefore statistically coherent with Eurostat crop acreage data. The ‘cell weights’ essentially indicate the UAA in a cell, corrected by a factor to ensure coherence between cell-level UAA (derived from the remote sensing-based 100-m CORINE Land Cover products) and the administrative regional UAA data from Eurostat. We capped those ‘cell weight’ values that exceed 100% at this maximum to be able to interpret them as UAA. UAA loss was under 5% for all countries during 2010–2015 while higher in 2016–2020, notably in Austria (30%), Belgium (17%), and Bulgaria (17%) (Supplementary Fig. S1). DGPCM UAA was calculated by multiplying the updated ‘weight’ by the grid cell area.

Additionally, some crop types in DGPCM were aggregated to align with crop classification in Eurostat_2010. Specifically, we modified the group of ‘other cereals’ to include all cereals except rice and maize; 8 crop types were merged into ‘other crops’, including root crop, soya, tobacco, other industrial crops, flowers, forage plants, nurseries, and other permanent crops (Table 3). In total, the ECIRA dataset includes 16 crop types: maize, rice, other cereals, pulses, potato, sugar beet, rapeseed (rape and turnip rape), sunflower, textile crops, (fresh) vegetable-melon-strawberry open field, grassland, fruit and berry, citrus, olive, vineyard, and other crops (Table 3).

**Table 3:** Crop classifications in ECIRA, DGPCM, and LUCAS datasets.

ECIRA code	ECIRA crop types (16)	DGPCM code	DGPCM crop types (28)	LUCAS codes and crop types
CERE	Other cereals (excluding maize and rice)	SWHE	Soft wheat	B11 Common wheat
		DWHE	Durum wheat	B12 Durum wheat
		BARL	Barley	B13 Barley
		RYEM	Rye	B14 Rye
		OATS	Oats	B15 Oats
		OCER	Other cereals	B18 Triticale; B19 Other cereals
LMAIZ	maize	LMAIZ	Maize	B16 Maize
PARI	rice	PARI	rice	B17 Rice
PULS	Pulses	PULS	Pulses	B41 Dry pulses
POTA	Potato	POTA	Potato	B21 Potatoes
SUGB	Sugar beet	SUGB	Sugar beet	B22 Sugar beet
SUNF	Sunflower	SUNF	Sunflower	B31 Sunflower
LRAPE	Rape and turnip rape	LRAPE	Rape and turnip rape	B32 Rape and turnip seeds
TEXT	Textile crops	TEXT	Textile crops	B34 Cotton; B35 Other fiber and oleaginous crops
GRAS	Grassland	GRAS	Grassland	E10 Grassland with sparse tree/shrub cover
				E20 Grassland without tree/shrub cover
				E30 Spontaneously vegetated surfaces
TOMA_OVEG	Fresh vegetable, melon, strawberry – open field	TOMA_OVEG	Fresh vegetable, melon, strawberry – open field	B42 Tomatoes; B43 Other fresh vegetables
				B45 Strawberry
APPL_OFRU	Fruit and berry	APPL_OFRU	Fruit and berry	B71 Apple; B72 Pear; B73 Cherry; B74 Nuts trees;
				B75 Other fruit trees and berries
CITR	Citrus	CITR	Citrus	B76 Oranges; B77 Other citrus fruit
OLIVGR	Olive	OLIVGR	Olive	B81 Olive groves
VINY	Vineyards	VINY	Vineyards	B82 Vineyard
OTHER	Other crops	ROOF	Root and forage crops	B23 Other root crops
		SOYA	Soya	B33 Soya
		TOBA	Tobacco	B36 Tobacco
		OIND	Other industrial crops	B37 Other non-permanent industrial crops
		FLOW	Flowers	B44 Floriculture and Ornamental plants
		OFAR	Forage plants	B51 Clovers; B52 Lucerne; B53 Other legumes and mixtures for fodder; B54 Mixed cereals for fodder; B55 Temporary grassland
		NURS	Nurseries	B83 Nurseries
		OCRO	Other permanent crops	B84 Permanent industrial crops

**Note:** ECIRA is the European crop-specific irrigated area dataset generated in this study; DGPCM is the Probabilistic Data Generating Process-based Crop Type Map^46^; LUCAS is the land use/cover area frame statistical survey dataset.

### Overview of the method

The development of the ECIRA dataset entails the following steps performed subsequently (Fig. 1):

(i) Disaggregate the annual total AAI (sourced from ELIAD) for the UK and Germany from the NUTS1 to NUTS2 level for 2010–2020, using the NUTS2-level sum of crop-specific AAI sourced from the Eurostat_2010.
(ii) Calculate NUTS2-level crop-specific AAI for each year by multiplying the total AAI from ELIAD with the proportion of crop-specific AAI to the sum of crop-specific AAI reported in Eurostat_2010.
(iii) Generate 1 km gridded total AEI for 2010 using the GMIA v5.0 (AEI for 2005 at 0.6 arc minutes) and HID 2010 (AEI for 2010 at 5 arc minutes), along with the bilinear spatial interpolation.
(iv) Calculate the annual 1 km gridded crop growing area by multiplying the UAA (updated ‘weight’ in the DGPCM dataset, ≤100%) with the crop growing shares for 2010–2020.
(v) At the 1 km grid level, multiply the AEI (result from Step iii) with the crop growing share, followed by aggregating this 1 km gridded crop-specific AEI at the NUTS2 level.
(vi) A calibration coefficient is calculated for each NUTS2 region, crop, and year as the ratio between the percentage of the crop-specific AAI (Step ii) to crop-specific AEI (Step v). This coefficient is then applied to the 1-km crop-specific AEI (Step v) to obtain the crop-specific AAI at 1 km. Finally, a post-processing step is performed to ensure that the total AAI does not exceed 100 ha, by applying a constraint coefficient to scale the values accordingly and produce the final 1-km gridded crop-specific AAI.

### Disaggregate total AAI from NUTS1 to NUTS2 level for the UK and Germany

Firstly, the total AAI data at the NUTS1 level provided by ELIAD (denoted as ${\widehat{TAAI}}_{{NUTS}1}$) for the UK and Germany were disaggregated to the NUTS2 level (Fig. 1). To differentiate between irrigated area data from ELIAD and Eurostat_2010, ELIAD data are denoted with a hat symbol, while Eurostat_2010 data are not. The subscripts NUTS1 and NUTS2 represent data or data processing at the NUTS1 and NUTS2 levels, respectively. This disaggregation assumed that the proportions of total AAI for each NUTS2 unit relative to the corresponding NUTS1 unit (*P_NUTS*) remained constant throughout the 2010–2020 period. The *P_NUTS* was computed using the NUTS2 and NUTS1 reported sum of crop-specific AAI from Eurostat_2010 (denoted as *TAAI*, Eq. 1). *TAAI*_*NUTS1*_ was calculated by summing up the corresponding *TAAI*_*NUTS2*_. When *TAAI*_*NUTS2*_ was not accessible for the UK, the ${\widehat{TAAI}}_{{NUTS}1}$ was disaggregated evenly to the corresponding NUTS2 units. The disaggregated NUTS2-level $\widehat{TAAI}$ was obtained using *P_NUTS* and ${\widehat{TAAI}}_{{NUTS}1}$ (Eq. 2).

1 $${P{\rm{\_}}{NUTS}}_{n,Y2010}( \% )=100\times \frac{{{TAAI}}_{{NUTS}2,n,Y2010}}{\,{\sum }_{n=1}^{N}{{TAAI}}_{{NUTS}2,n,Y2010}}$$

2 $${\widehat{{T}{A}{A}{I}}}_{{NUTS}2,n,{YA}}={P{\rm{\_}}{NUTS}}_{n,Y2010}/100\times {\widehat{{TAAI}}}_{{NUTS}1,{YA}}$$

Where the subscript Y2010 indicates that data is temporally fixed for 2010, while YA denotes an annual update; *n* represents a specific NUTS2 unit; and *N* is the total number of NUTS2 units within a specific NUTS1 unit.

### Generating NUTS2 level crop-specific AAI for 2010–2020

The next step is disaggregating annual ${\widehat{TAAI}}_{{NUTS}2}$ to different crops (Fig. 1). The percentage of AAI for each crop to *TAAI* (denoted as *P_crop*) was computed as follows,

3 $${P{\rm{\_}}{crop}}_{{NUTS}2,c,Y2010}( \% )=100\times \frac{{{AAI}}_{{NUTS}2,c,Y2010}}{{\sum }_{c=1}^{C}{{AAI}}_{{NUTS}2,c,Y2010}}$$

Where *AAI*_*NUTS2,c,Y2010*_ represents the irrigated area of crop *c* at the NUTS2 level, temporally fixed for 2010, from the Eurostat_2010 dataset. *C* is the total number of crop types in ECIRA (*C* = 16). During 2010–2020, the reported crop-specific AAI was only accessible in 2010. Therefore, we assumed that the *P_crop*_*NUTS2,c*_ remained consistent throughout 2010–2020; namely *P_crop*_*NUTS2,c,Y2010*_ was utilized, which is temporally fixed for 2010. The annual NUTS2 level crop-specific AAI (${\widehat{AAI}}_{{NUTS}2,c,{YA}}$) was estimated using Eq. 4. However, it is noteworthy that $\widehat{TAAI}$ differs from the reported total AAI and the sum of crop AAI in Eurostat_2010, as $\widehat{TAAI}$ sourced from the ELIAD accounts for the corrected irrigation reference period, the availability of irrigation infrastructure, and the impacts of climate variability on irrigation percentage.

4 $${\widehat{AAI}}_{{NUTS}2,c,{YA}}={P{\rm{\_}}{crop}}_{{NUTS}2,c,Y2010}/100\times {\widehat{TAAI}}_{{NUTS}2,{YA}}$$

### Generating total AEI percentage at 1 km grid for the reference year 2010

Given that the prerequisite for irrigation is the availability of irrigation infrastructure, the development of ECIRA accounted for the spatial patterns of the total AEI. To generate the total AEI (TAEI) for 2010 at 1 km resolution (denoted as *TAEI*_*1km,Y2010*_), data from GMIA v5.0^32^ for 2005 at 0.6 arc min resolution across Europe (*TAEI*_*0.6min,Y2005*_) and HID^35^ in 2010 at 5 arc minute resolution (*TAEI*_*5min,Y2010*_) were combined (Fig. 1 and Table 2). Firstly, the sum of *TAEI*_*0.6min,Y2005*_ grids within each 5-arc min grid was calculated as *TAEI*_*5min,Y2005*_. Next, at the 5-arc min level, the percentage of *TAEI*_*5min,Y2005*_ to *TAEI*_*5min,Y2010*_ was determined (*P_TAEI*_*5min*_, Eq. 5), and was resampled to 1 km (*P_TAEI*_*1km*_) using the Bilinear interpolation with the EPSG 3035 projection. This interpolation calculated the weighted average of the four nearest input grid cell values, providing smooth transitions between grids. Finally, *TAEI*_*0.6min,Y2005*_ was resampled to 1 km (*TAEI*_*1km,Y2005*_) using the Bilinear interpolation, and multiplied with *P_TAEI*_*1km*_ to get the estimated total AEI in 2010 at 1 km resolution (Eq. 6).

5 $${P{\rm{\_}}{TAEI}}_{5\min }( \% )=100\times \frac{\,{{TAEI}}_{5\min ,Y2005}}{{{TAEI}}_{5\min ,Y2010}}$$

6 $${{TAEI}}_{1{km},Y2010}={P{\rm{\_}}{TAEI}}_{1{km}}/100\times {{TAEI}}_{1{km},Y2005}$$

### Determining 1 km-gridded crop-specific irrigated area

At the 1 km grid level (Fig. 1), the UAA (calculated as the updated ‘cell weights’ multiplied by the 1 km grid area) was multiplied by the crop growing share (CGS) from the DGPCM to obtain the crop-specific growing area. The crop growing share was then multiplied by the total AEI to get the crop-specific AEI (Eq. 7), which revealed the spatial distributions of irrigation infrastructure for different crops. The crop-specific AEI for all 1 km grid cells within each NUTS2 unit were then aggregated to obtain the NUTS2 level crop-specific AEI (*AEI*_*NUTS2,C,YA*_). Subsequently, at the NUTS2 level, the percentage of estimated crop-specific AAI (${\widehat{AAI}}_{{NUTS}2,c,{YA}}$) to crop-specific AEI was calculated to get the calibration coefficient (denoted as *P_AAI*_*c,YA*_, Eq. 8) for each NUTS2 unit. All 1-km grid cells within the same NUTS2 unit were assigned the same calibration coefficient. Then the 1-km gridded calibration coefficient was multiplied by the crop-specific AEI grids to obtain the 1-km gridded crop-specific AAI (Eq. 9).

7 $${{AEI}}_{1{km},c,{YA}}={{CGS}}_{1{km},c,{YA}}\times {{TAEI}}_{1{km},Y2010}$$

8 $${P{\rm{\_}}AAI}_{c,{YA}}( \% )=100\times \frac{{\widehat{{AAI}}}_{{NUTS}2,c,{YA}}}{{{AEI}}_{{NUT}2,c,{YA}}}$$

9 $${{AAI}}_{1{km},c,{YA}}={{AEI}}_{1{km},c,{YA}}\times {P{\rm{\_}}{AAI}}_{c,{YA}}/100$$

Finally, when the total crop-specific AAI exceeds 100 ha, a uniform scaling factor is applied to proportionally reduce the values, ensuring the total equals 100 ha and yielding the final crop-specific AAI at 1-km resolution. The rainfed area is subsequently derived by subtracting the irrigated area from the crop growing area.

## Data Records

The 1 km gridded European Crop-specific IRrigation Area (ECIRA) dataset is available at Zenodo^52^. ECIRA provides annual total and crop-specific irrigated and rainfed area across 28 European countries for 2010–2020 (11 years). The sum of irrigated and rainfed area equals the crop growing area. ECIRA includes 16 crop types: maize, rice, other cereals (excluding maize and rice), pulses, potato, sugar beet, rapeseed (rape and turnip rape), sunflower, textile crops, fresh vegetable & melon & strawberry in open fields, grassland, fruit and berry, citrus, olive, vineyard, and other crops. Total irrigated (rainfed) area is the sum of irrigated (rainfed) area for all crop types. The 28 European countries include Austria, Belgium, Bulgaria, Cyprus, the Czech Republic, Germany, Denmark, Estonia, Greece, Spain, Finland, France, Croatia, Hungary, Ireland, Italy, Lithuania, Luxembourg, Latvia, Malta, Netherlands, Poland, Portugal, Romania, Sweden, Slovenia, Slovakia, and the United Kingdom. The abbreviation of the countries and subnational regions adopts the NUTS (Nomenclature of Territorial Units for Statistics) standard (Supplementary Table 1).

ECIRA data is numeric (float), with values representing hectares, ranging from 0 to 100 for each 1 km grid cell, in *GeoTIFF* format using the EPSG 3035 projection. Each *GeoTIFF* records the crop-specific (or total) irrigated (or rainfed) area for a specific year. The naming format for each *GeoTIFF* file is ‘*Crop type – irrigation – year. tif*’. The naming code for each crop type is shown in Table 3 (the ‘ECIRA code’ column). The irrigation is identified by *IR* (irrigated) and *RF* (rainfed). For example, the ‘*LMAIZ_IR_A_2010.tif*’ represents the growing area of irrigated maize in the year 2010; ‘*LMAIZ _RF_A_2010.tif*’ represents the growing area of rainfed maize in the year 2010; ‘*LMAIZ_A_2010.tif*’ represents the total maize growing area in 2010, which is the sum of the irrigated and rainfed maize areas, aligning with the crop growing area calculated using crop growing share and updated ‘cell weights’ from the DGPCM dataset. ‘*Total_IR_A_2015.tif*’ and ‘*Total_RF_A_2015.tif*’ represent the total irrigated area (sum of irrigated area for all crops) and total rainfed area (sum of rainfed area for all crops) in 2015, respectively. ‘*UAA_2010.tif*’ represents the utilized agricultural area in 2010, which is the sum of total irrigated and rainfed areas.

## Technical Validation

Irrigation data comparison focuses on crop-specific irrigated hotspots (areas with high irrigation concentration) using three datasets - the Land Use/ Cover Area Frame Statistical Survey (LUCAS) irrigated points with 16 crop types in 2012, 2015, and 2018^53^, the Spatial Production Allocation Model irrigation area with 12 crop types in 2010 (SPAM 2010)^33^, and the European irrigation area map in 2010 with 14 crop types (EIM 2010)^3^.

LUCAS consists of *in-situ* surveys conducted through stratified field investigations within a 2 km grid across parts of European countries^53^. Some points that are either unlikely to change or are challenging to access are classified through photo interpretation, utilizing the latest ortho-photos or very high-resolution imagery^53^. This point-level data provides land cover and land use information from 2006 to 2018, updated every three years. Irrigation information is available in 2012, 2015, and 2018 for the 2010–2020 period. Firstly, LUCAS points related to agricultural land use were filtered by examining the LU1 (Primary land use) or LU2 (Secondary land use) series labeled as agriculture (code with U111) and fallow land (U112). Subsequently, LUCAS points were divided into 16 crop types (Table 2) and irrigation management practices were identified using the water management (WM) label – irrigation (WM 1), potential irrigation (WM 2), drainage (WM 3), irrigation and drainage (WM 4), no visible water management (WM 5), and not relevant (WM 8). Points with WM labels 1 and 4 were identified as irrigated points that were receiving or had received irrigation during the LUCAS investigation.

Given the point-based nature of LUCAS data and the limited number of irrigation observations (fewer than 1000 per crop type across the study region), we conducted a quantitative comparison of irrigated percentage (denoted as IP, Eqs. 10 and 11) between the LUCAS and ECIRA data at the 40 km grid level, incorporating data from years 2012, 2015, and 2018.

10 $${{IP}}_{{LUCAS}}( \% )=100\times {NI}/{NT}$$

11 $${{IP}}_{{ECIRA}}\,( \% )=100\times {TAAI}/{TA}$$

Where *NI* and *NT* are the number of irrigated and total investigated points within a grid, respectively; TAAI and *TA* are the total irrigated area and the grid area, respectively. Grids with irrigated percentages below 0.3% were excluded due to the high omission probability by LUCAS in areas with very low irrigation. We selected eight crop types for grid-wise comparison: maize, other cereals, citrus, fruit and berries, olive, vegetables & strawberries & melons, vineyards, and rice, each with a sample size greater than 150, except for rice, which was included despite having a smaller sample size, as it is generally irrigated. In addition, we performed visual comparisons between the crop-specific ECIRA irrigated area and the crop-specific hotspots of the LUCAS irrigated points. This visual comparison focused mostly on LUCAS 2018 (encompassing 28 European countries), because the year 2018 is particularly dry in Europe and we expect irrigation practices to be more detectable during the on-site single survey compared to other wetter years.

The EIM 2010 dataset provides crop-specific and total AAI across the European Union Member States and the UK for the reference year of 2010^3^. EIM 2010 contains 14 irrigated crop types (Table 4), with a spatial resolution of 10 km. The primary input data for generating EIM 2010 is from the Eurostat 2010 Agricultural Census, including subnational crop-specific AAI, 10-km gridded total crop growing area, and total AAI. A custom-developed strategy incorporating expert judgment was employed to disaggregate subnational crop-specific irrigated area into 10 km grids. The crop type classification of EIM 2010 and our ECIRA is consistent because both are sourced from the Eurostat-reported data.

**Table 4:** Crop classifications in ECIRA, SPAM, MIRCA-OS, and EIM2010 datasets.

ECIRA crop types (16)	EIM 2010 crop types (14)	ECIRA aggregated crop types (12)	SPAM 2010 crop types (11)	MIRCA-OS 2010 crop types (11)
Other cereals excluding maize & rice	Cereals	Other cereals excluding maize & rice	Wheat, barley, pearl millet, small millet, sorghum, other cereals	Barley, millet, wheat, sorghum, rye
maize	Maize	Maize	Maize	Maize
rice	Rice	Rice	Rice	Rice
Pulses	Pulses	Pulses	Other pulses	Pulses
			Groundnut	Groundnuts
Potato	Potato	Potato	Potato	Potato
Sugar beet	Sugar beet	Sugar beet	Sugar beet	Sugar beet
Sunflower	Sunflower	Sunflower	Sunflower	Sunflower
Rape and turnip rape	Turnip rape	Rape and turnip rape	Rapeseed	Rapeseed
Textile crops		Textile crops	Cotton	Cotton
			Other fibre crops	
Grassland	Grassland	Grassland	—	—
Fresh vegetable, melon, strawberry – open field	Fresh vegetable, melon, strawberry – open field	Vegetable and fruit	Vegetables	Other perennial
			Banana	
			Plantain	
			Tropical fruit	
			Temperate fruit	
Fruit and berry	Fruit and berry			
			Coconut	
Citrus	Citrus			
Olive	Olive			
Vineyards	Vineyards			
Other crops		Other crops	Sweet potato, yams, cassava, other roots, bean, chickpea, cowpea, pigeon pea, lentil, soya bean, oil palm, seam seed, other oil crops, sugarcane, Arabica coffee, Robusta coffee, cocoa, tea, tobacco, rest of crops	Cassava, cocoa, coffee, fodder, soybean, sugarcane, oil palm, other annual

Note: EIM 2010 is the European irrigation area map for the year 2010; SPAM 2010 is the spatial production allocation model dataset for the reference year of 2010; MIRCA-OS 2010 is the MIRCA open-source dataset for the reference year of 2010. For comparing SPAM 2010 and ECIRA datasets, we aggregated some crop types in ECIRA data, and these aggregated crop types are listed in the ‘ECIRA aggregated crop types’.

The SPAM dataset provides global crop-specific harvest areas by identifying different farming systems based on agricultural inputs such as machinery, labor, nutrients, and chemical disease and weed control^33^. These systems include irrigated, rainfed high-input, rainfed low-input, and rainfed subsistence farming systems. However, it should be noted that the ‘irrigated area’ in SPAM is the irrigable area, because SPAM utilizes the GMIA v5.0 dataset which is global AEI data at a 5-arc minute resolution, with a fixed reference year of 2005^32^. The primary data source for SPAM is national statistical crop harvest area data, and the generation of SPAM minimizes errors in area allocation within each grid cell using a cross-entropy module. The SPAM data is updated for the years 2000, 2005, 2010, 2017, and 2020, with a spatial resolution of 5 arc minutes. However, SPAM 2017 only covers Sub-Saharan Africa^54^, and SPAM 2020 exhibits the same irrigation hotspots as SPAM 2010, because both of them use irrigation area data sourced from the GMIA v5.0. Therefore, only the SPAM 2010 encompassing 42 crops was utilized for data comparison. Some crop types were aggregated to align them into 12 types for comparison with the ECIRA dataset (Table 4). The comparison between SPAM 2010 and ECIRA focused on six crop types that are either widely cultivated or have high irrigation requirements, including maize, rice, other cereals (excluding maize and rice), vegetables and fruit, potato, and sugar beet.

The MIRCA-OS dataset^39,40^ provides global monthly crop-specific irrigated area data for 2000, 2005, 2010, and 2015 at 5 arc minutes resolution, covering 23 crop types. For European countries, NUTS2-level irrigation data were sourced from Eurostat. Eurostat crop irrigation data are available from 2000 to 2013 but are sparse for most countries, except for 2010, which has complete coverage. When data for a specific year were unavailable, national-level irrigation estimates from AQUASTAT were disaggregated to NUTS2 units, assuming constant regional proportions. For years with multiple data points, linear interpolation was applied to estimate crop-specific irrigated areas. Due to data limitations and uncertainties in disaggregation and interpolation, only the 2010 dataset was used for comparison with ECIRA. Furthermore, given that MIRCA-OS data are monthly and irrigated double cropping in Europe hardly exists, the maximum monthly growing area grids from MIRCA-OS were selected for the comparison. Due to differences and overlaps in crop classifications between MIRCA-OS and ECIRA (Table 4), only 8 crop types were selected: maize, rice, other cereals, pulses, potato, sugar beet, sunflower, and rapeseed.

We also performed a quantitative comparison between ECIRA and other crop-specific irrigation datasets. Given the differences in spatial resolutions (SPAM and MIRCA-OS at 5 arc-minute resolution, EIM at 10 km, and ECIRA at 1 km) and projections, the comparison was conducted at the 10 km grid level with EPSG 3035 projection. Specifically, the 1 km gridded ECIRA data were aggregated to 10 km by summing the values within each 10 km grid. The 5 arc-minute irrigation percentage (%) for the SPAM and MIRCA-OS datasets was calculated as the ratio of irrigated area to total area for each grid, and then resampled to 10 km. Additionally, following the data processing approach used for LUCAS, only grids identified as irrigated in both datasets with an irrigated percentage greater than 0.3% were retained for comparison.

### Comparison to LUCAS irrigation hotspots

**Fig. 2:**
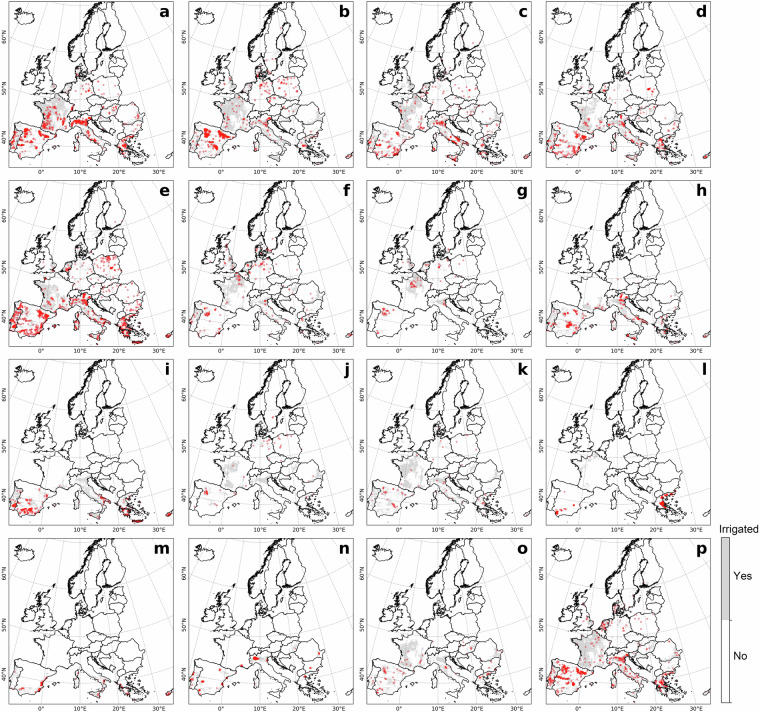
Crop-specific irrigated points in the LUCAS dataset in 2018. (**a**) Maize, (**b**) other cereals (excluding maize and rice), (**c**) vegetables, strawberry, and melon in open field, (**d**) fruit and berry, (**e**) grassland, (**f**) potato, (**g**) sugar beet, (**h**) vineyard, (**i**) olive, (**j**) rapeseed, (**k**) pulses, (**l**) textile crops, (**m**) citrus, (**n**) rice, (**o**) sunflower, and (**p**) other crops. The red points are irrigated points with LUCAS water management labels of ‘irrigation’ and ‘potential irrigation’; the light grey background is the irrigated area (>0 ha) identified in ECIRA; Crop type classifications are shown in Table 3. For other years see the Supplementary Figs. S2–S5.

**Fig. 3:**
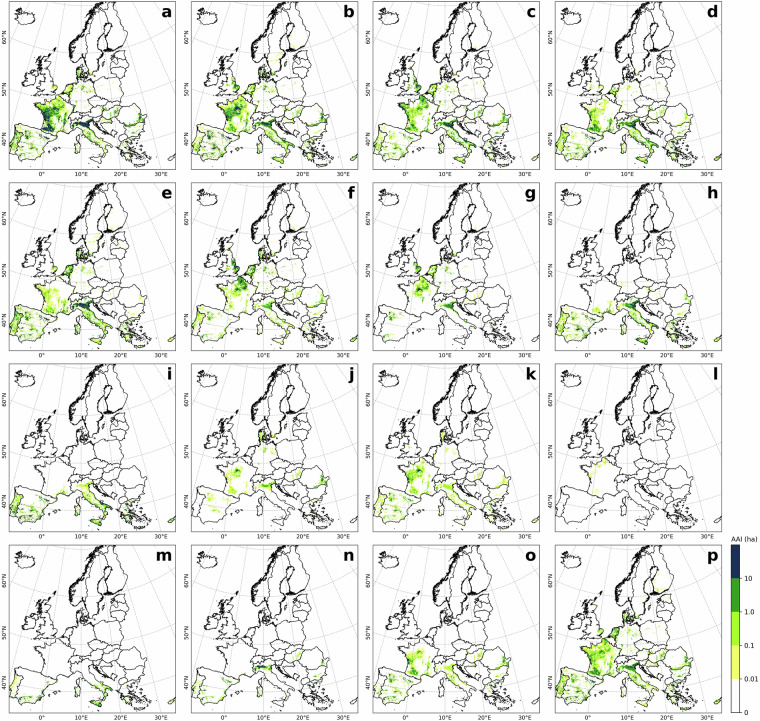
Crop-specific irrigated area in the ECIRA dataset in 2018 at 1 km grid level. (**a**) Maize, (**b**) other cereals (excluding maize and rice), (**c**) vegetables, strawberry, and melon in open field, (**d**) fruit and berry, (**e**) grassland, (**f**) potato, (**g**) sugar beet, (**h**) vineyard, (**i**) olive, (**j**) rapeseed, (**k**) pulses, (**l**) textile crops, (**m**) citrus, (**n**) rice, (**o**) sunflower, and (**p**) other crops. Crop type classification is shown in Table 3.

**Fig. 4:**
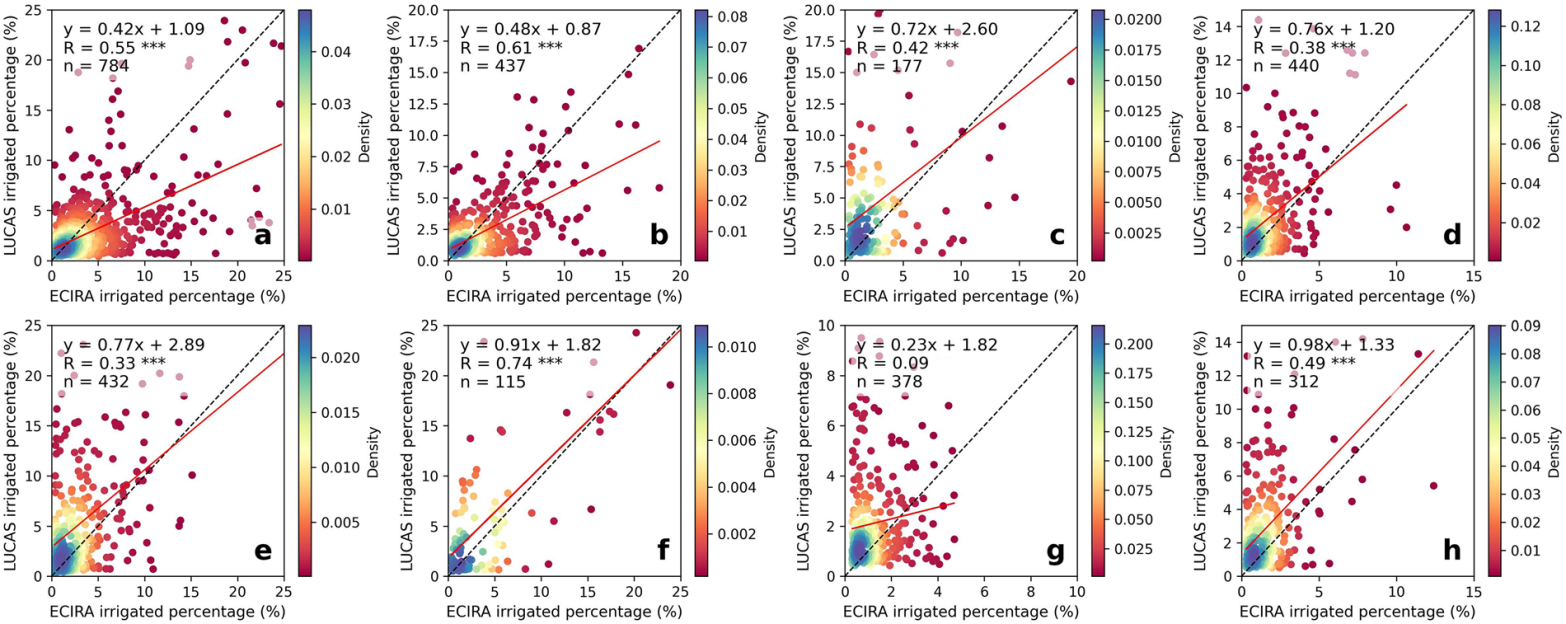
Comparison of ECIRA and LUCAS irrigated percentage at 40 km grid level. (**a**) Maize, (**b**) other cereals (excluding maize and rice), (**c**) citrus, (**d**) fruit & berry, (**e**) olive, (**f**) rice, (**g**) vegetables & strawberry & melon, and (**h**) vineyard. Data for years 2012, 2015, and 2018 are combined; the red line is the fitted linear regression function and the black dash is the 1:1 line. The color bar shows the Gaussian kernel density values, representing the estimated probability density at a given point, with high values indicating high concentrations of data points. Grids with irrigated percentages lower than 0.30% were not included in this comparison. Statistical significance is indicated by asterisks: ***p < 0.001, **p < 0.01, *p < 0.05, and no asterisk denotes *p* > 0.05.

Overall, crop-specific irrigated hotspots identified by LUCAS and ECIRA were highly consistent in 2018 (Figs. 2, 3), and similar results were observed in 2012 and 2015 (Supplementary Figs. S2–S5). Both ECIRA and LUCAS highlighted irrigation hotspots in the Iberian Peninsula, western France, the Apennine Peninsula (particularly south of the Alps), and the southern and eastern Balkans. However, ECIRA identified irrigated hotspots in some temperate and relatively humid regions that were not detected by LUCAS 2018. For example, LUCAS 2018 did not fully detect irrigated areas for other cereals in western France and eastern Romania, vegetables & strawberries & melons in eastern Britain and eastern Romania, and grassland in the Jutland Peninsula (Figs. 2, 3). LUCAS 2018 identified substantial irrigation in Poland for other cereals and grasslands, which was not detected by ECIRA (Figs. 2, 3). However, LUCAS 2012 and 2015 did not identify such substantial irrigation for grassland and other cereals in Poland (Figs. S3, S5). For the grid-wise comparison at the 40 km level, the agreement between ECIRA and LUCAS irrigation percentages was relatively strong (R ≥ 0.42) for maize, rice, other cereals, citrus, and vineyards, but weaker for fruit & berries, olives, and vegetables & strawberries & melons (Fig. 4). Furthermore, the slopes of the linear regressions were generally below 0.80 (except for rice and vineyard), suggesting that LUCAS tends to estimate lower irrigation percentages compared to ECIRA.

LUCAS data were collected at specific points within a 2 km grid at particular times of the year^42^. Therefore, misalignment of survey timing with irrigation activities or non-representative observation locations can result in the overlook of irrigation. In humid regions of Europe, irrigation supplements precipitation to ensure normal crop growth^3,55^, making it challenging to capture this practice through a single on-site survey. In contrast, ECIRA primarily relies on irrigation data from farm structure surveys and agricultural censuses that report irrigation over the past 12 months^14^, thereby reducing the possibility of overlooking irrigation practices. This can explain why ECIRA detected more irrigation in humid and temperate regions than LUCAS. However, LUCAS 2018 identified substantial irrigation in Poland, while ECIRA did not, and LUCAS 2012 and 2015 did not show this pattern either. This inconsistency is difficult to explain due to the lack of additional information. A possible reason could be uncertainties in the LUCAS 2018 data collection for Poland, such as inaccuracies in defining irrigated areas and the poor representativeness of the sampling points. Another potential reason could be a mismatch of definitions, since a large part of the former irrigation infrastructure in Poland has been in ameliorated wetlands equipped for combined drainage and irrigation^56^ and used as grassland. For surveyors visiting the sites, it might therefore be difficult to decide whether the remaining infrastructure is actually used for drainage, irrigation, or both. The inconsistency between ECIRA and LUCAS 2018 in identifying irrigated areas for textile crops (Figs. 2I, 3I) could be attributed to the limited data available for these crops, which have very small cultivation areas and encompass a wide variety of species. Moreover, maize, rice, other cereals, citrus, and vineyards exhibited stronger grid-wise agreement between ECIRA and LUCAS, possibly owing to their concentrated cultivation and high irrigation demands. In contrast, for crops with more complex irrigation patterns, such as fruits and vegetables, LUCAS and ECIRA may face challenges in capturing irrigation data, particularly when these crops are more dispersed or grown in smaller areas.

### Comparison to SPAM 2010 irrigation area

**Fig. 5:**
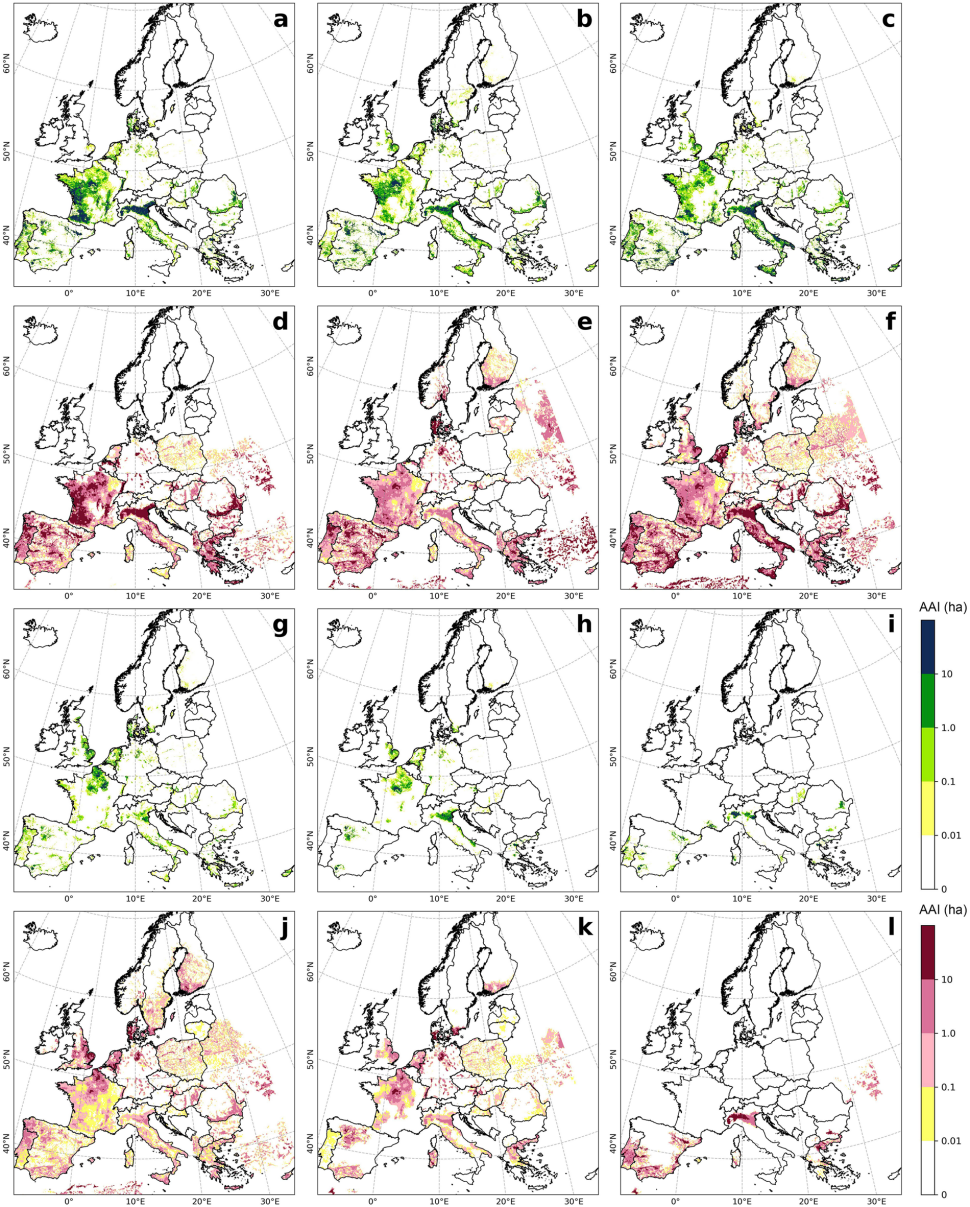
Crop-specific irrigation area in ECIRA and SPAM datasets in 2010. ECIRA data: (**a**) maize, (**b**) other cereals (excluding maize and rice), (**c**) vegetable & fruit, (**g**) potato, (**h**) sugar beet, and (**i**) rice. SPAM data: (**d**) maize, (**e**) other cereals (excluding maize and rice), (**f**) vegetable & fruit, (**j**) potato, (**k**) sugar beet, and (**l**) rice. Specific crop type classification is shown in Table 4. Other crop types are shown in Supplementary Fig. S6.

**Fig. 6:**
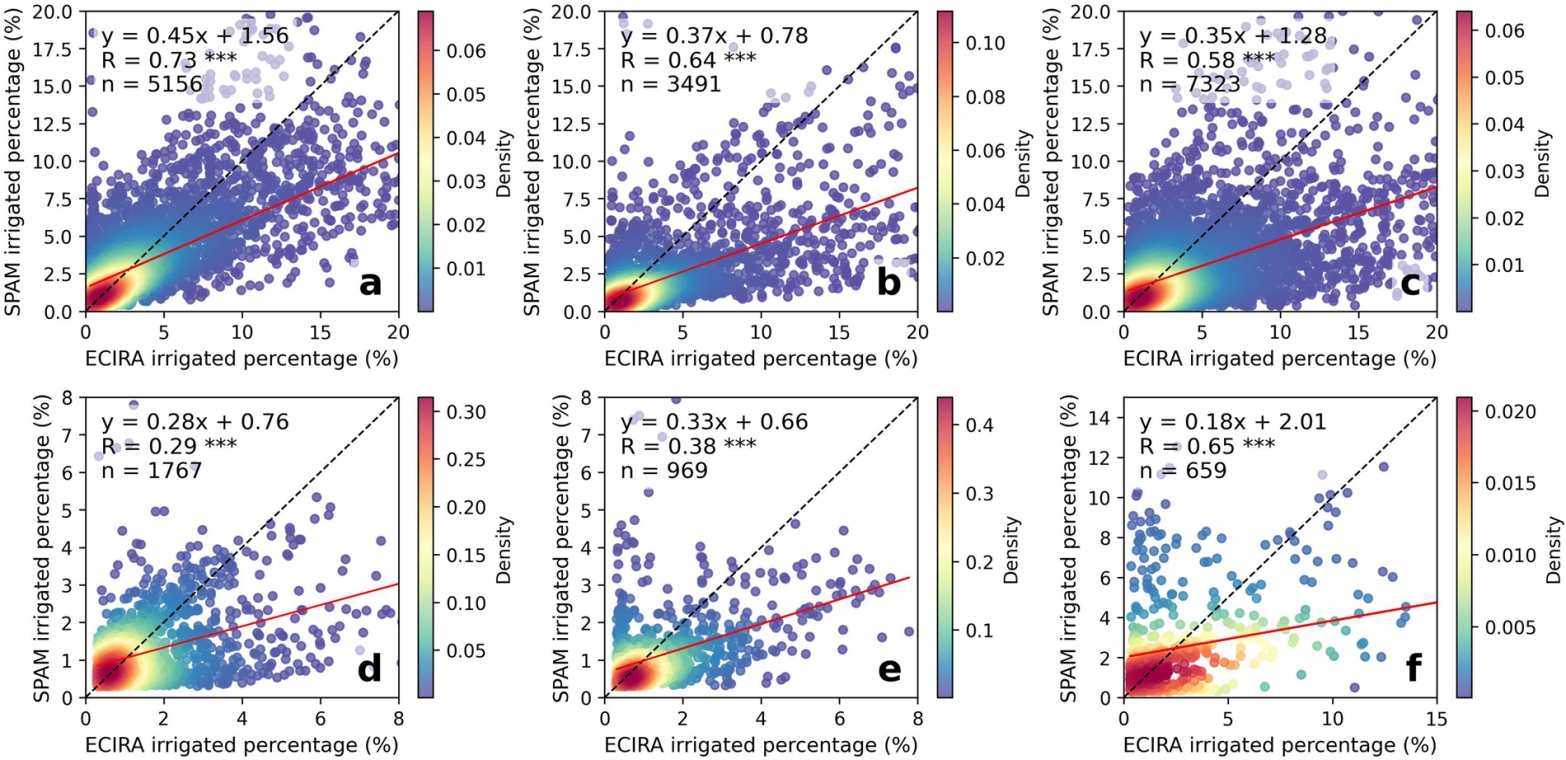
Grid-wise comparison of ECIRA and SPAM 2010 irrigated percentage at 10 km level. (**a**) Maize, (**b**) other cereals, (**c**) vegetables and fruit, (**d**) potato, (**e**) sugar beet, and (**f**) rice. Grids with irrigated percentages lower than 0.30% were not included in this comparison.

In southern EU regions characterized by extensive irrigation, the SPAM 2010 identified a larger and more widespread crop-specific irrigation area than ECIRA, particularly in the Iberian Peninsula, southern Balkan Peninsula, and western France (Fig. 5). In western EU regions, it was challenging to conclude whether ECIRA or SPAM 2010 detects a more extensive crop-specific irrigation area (Fig. 5). For instance, ECIRA reported higher and broader irrigation areas for other cereals and maize in the Netherlands and eastern Great Britain, while SPAM 2010 reported wider irrigation areas for vegetable and fruit as well as potato in these regions. In northern EU regions, excluding the Jutland Peninsula, SPAM 2010 generally reported higher irrigation areas than ECIRA (Fig. 5). Similarly, in eastern and central EU regions, SPAM 2010 detected more widespread and larger irrigation areas than ECIRA, except for the other cereals and rice in eastern Romania (Fig. 5). For the other crop types with smaller cultivation areas, such as pulses and rapeseed in western France (Supplementary Fig. S6), the consistency between SPAM 2010 and ECIRA was relatively weaker than the main crops. Nevertheless, both ECIRA and SPAM 2010 effectively identified crop-specific irrigation hotspots and generally showed good consistency in spatial patterns for extensively cultivated and irrigated crops. However, ECIRA typically reported fewer irrigation areas compared to SPAM 2010, particularly in regions with extensive irrigation. For the 10 km grid-wise comparison (Fig. 6), maize, rice, and other cereals showed strong agreement between SPAM 2010 and ECIRA (R ≥ 0.64), while the agreement was weaker for potatoes and sugar beet, similar to the findings in the comparison with LUCAS. Additionally, although the slopes of the linear regressions were below 1.0, it cannot be concluded that ECIRA reported higher irrigation areas than SPAM, as we only retained grids that were identified as irrigated by both SPAM and ECIRA.

The total irrigation and crop growing areas are two critical data sources for developing crop-specific irrigation area datasets. For ECIRA, these data were obtained from ELIAD (annual AAI mainly at NUTS2), GMIA v5.0 (AEI in 2005 at 0.6 arc minute resolution), and HID 2010 (AEI in 2010 at 5 arc minute resolution) for irrigation, along with the DGPCM dataset for crop growing areas. In contrast, SPAM 2010 derived its data from GMIA v5.0 (AEI in 2005 at 5 arc minute resolution) and subnational crop growing area which is primarily from Eurostat and FAOSTAT^33^. Cropland without irrigation infrastructure cannot be irrigated, indicating that AAI should not exceed AEI. SPAM 2010 represents the crop-specific cultivation extent equipped for irrigation, regardless of whether irrigation is applied, while ECIRA reflects the crop-specific areas actually irrigated. Additionally, AEI exhibits a decreasing trend from 2005 to 2010^26^, so the use of AEI from 2005 to reflect irrigation in 2010 can lead to an overestimation of crop-specific irrigation area in SPAM 2010. These factors could contribute to SPAM 2010 identifying a larger crop-specific irrigation area compared to ECIRA.

Regarding crop growing area, both SPAM 2010 and ECIRA derived their regional crop areas primarily from Eurostat surveys, contributing to the overall good consistency between them. However, at the grid cell level, SPAM 2010 and DGPCM (used as input for ECIRA) differed in spatial resolution, input data, and applied methodologies. For example, DGPCM was specifically developed for Europe using country-specific training data, whereas SPAM 2010 is a global model with a much lower spatial resolution and a more generalist spatial crop allocation procedure. Those differences contribute to differences in cell-level crop growing areas and, consequently, crop-specific irrigation area estimates between SPAM 2010 and ECIRA. Furthermore, despite efforts to align crop type classifications, the differing original classifications—16 crop types for ECIRA and 42 crop types for SPAM 2010—can introduce uncertainties in crop type aggregation and subsequent irrigation disaggregation.

### Comparison to MIRCA-OS 2010 irrigated area

**Fig. 7:**
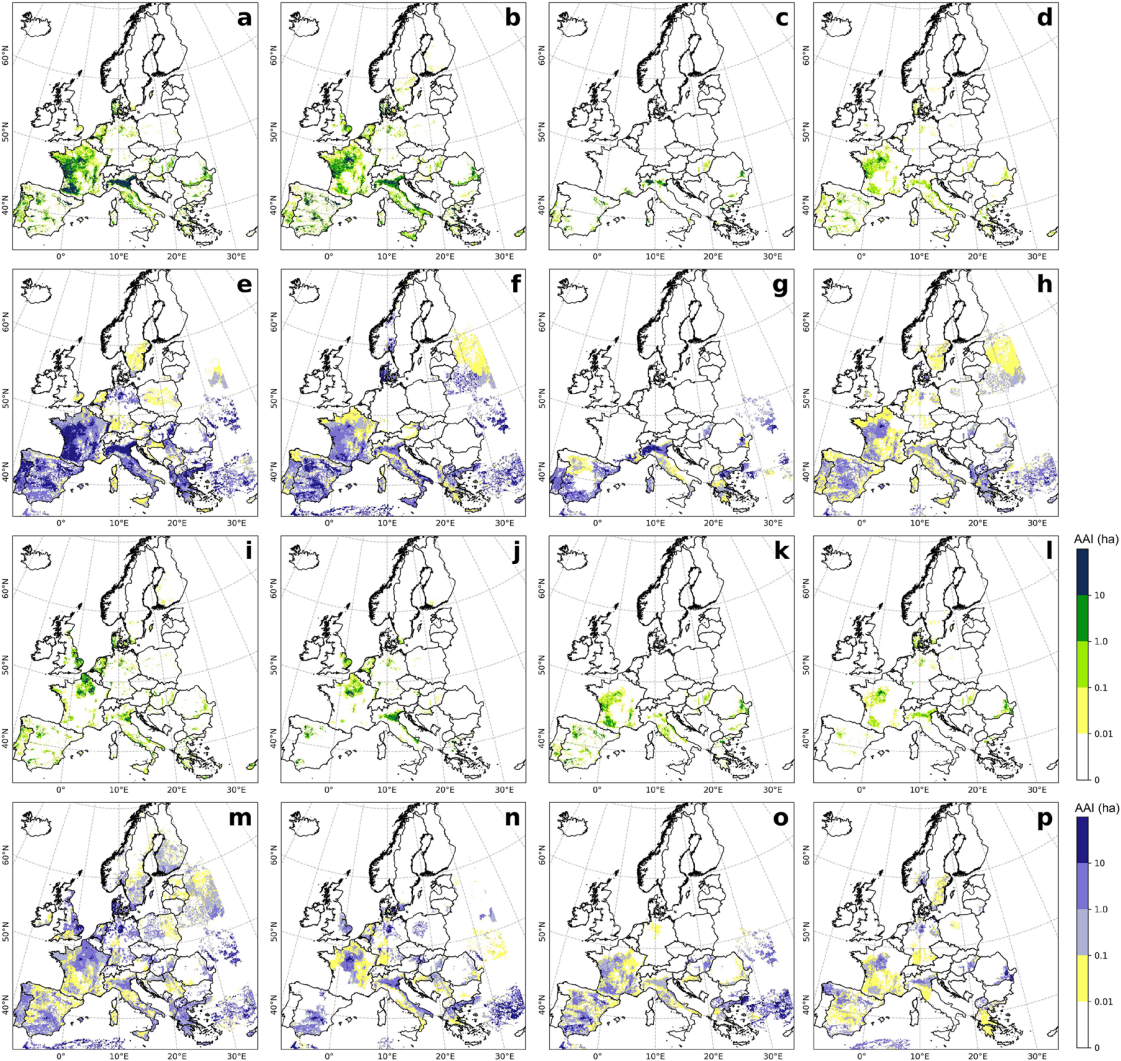
Crop-specific irrigated area in ECIRA and MIRCA-OS datasets in 2010 at 1 km grid level. ECIRA data: (**a**) maize, (**b**) other cereals (excluding maize and rice), (**c**) rice, (**d**) pulses, **(i**) potato, (**j**) sugar beet, (**k**) sunflower, and (**l**) rapeseed. MIRCA-OS data: (**e**) maize, (**f**) other cereals (excluding maize and rice), (**g**) rice, (**h**) pulses, (**m**) potato, (**n**) sugar beet, (**o**) sunflower, and (**p**) rapeseed. Specific crop type classification is shown in Table 4.

**Fig. 8:**
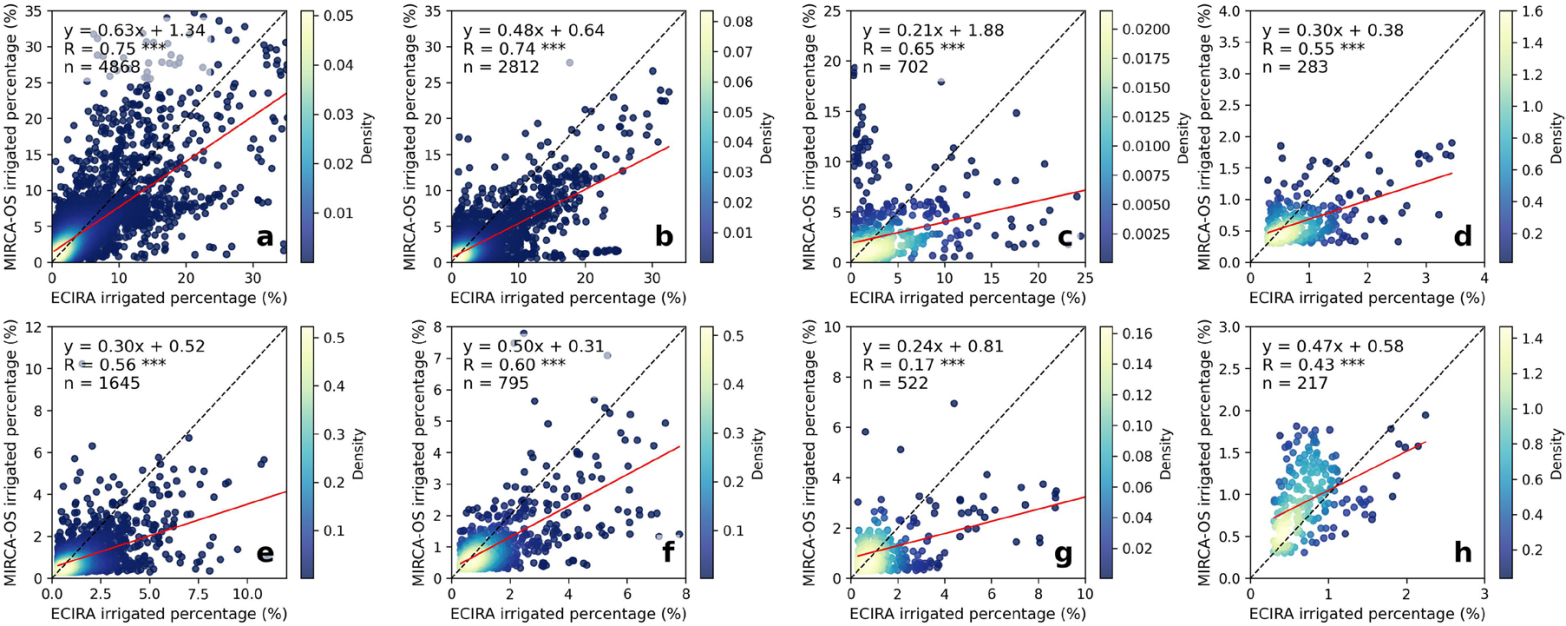
Grid-wise comparison of ECIRA and MIRCA-OS 2010 irrigated percentage at 10 km level. (**a**) Maize, (**b**) other cereals (excluding maize and rice), (**c**) rice, (**d**) pulses, (**e**) potato, (**f**) sugar beet, (**g**) sunflower, and (**h**) rapeseed. Grids with irrigated percentages lower than 0.30% were not included in this comparison.

The comparison between MIRCA-OS and ECIRA for 2010 (Fig. 7) showed high consistency in identifying irrigated hotspots, but ECIRA generally detected more concentrated irrigated areas than MIRCA-OS, which could be owing to the coarser spatial resolution of MIRCA-OS (5 arc minutes versus 1 km). Differences were mainly observed in the Iberian Peninsula: sugar beet irrigation was identified in the southern region by MIRCA-OS and in the northwest by ECIRA; sunflower irrigation was detected in ECIRA but not in MIRCA-OS for Portugal; and rapeseed was more widespread in MIRCA-OS than ECIRA. MIRCA-OS also missed irrigated areas in the Netherlands and eastern Great Britain for other cereals, likely due to differences in crop classification. Besides, the crop-specific scatter plot comparisons (Fig. 8) showed a strong agreement between ECIRA and MIRCA-OS datasets, as the correlation coefficients for most crops, excluding sunflower and rapeseed which had relatively smaller irrigated areas, ranged from 0.55 to 0.75, particularly high for maize and other cereals.

The high consistency between MIRCA-OS and ECIRA can be attributed to both datasets using Eurostat 2010 crop-specific irrigated area as their main data source. However, key differences in the methodology used for spatial disaggregation might contribute to the observed discrepancies. Specifically, MIRCA-OS considered multiple growing seasons for crops and employed the History Database of the Global Environment dataset for its cropland mask^40^. In contrast, our ECIRA utilized DGPCM as its land cover and crop-type mask. Besides, MIRCA-OS used a stepwise prioritization strategy during the disaggregation process, with an emphasis on crop priority. Another distinction was that ECIRA corrected the irrigation reference year, ensuring that the most accurate and up-to-date irrigation data were used.

### Comparison to EIM 2010 irrigated area

**Fig. 9:**
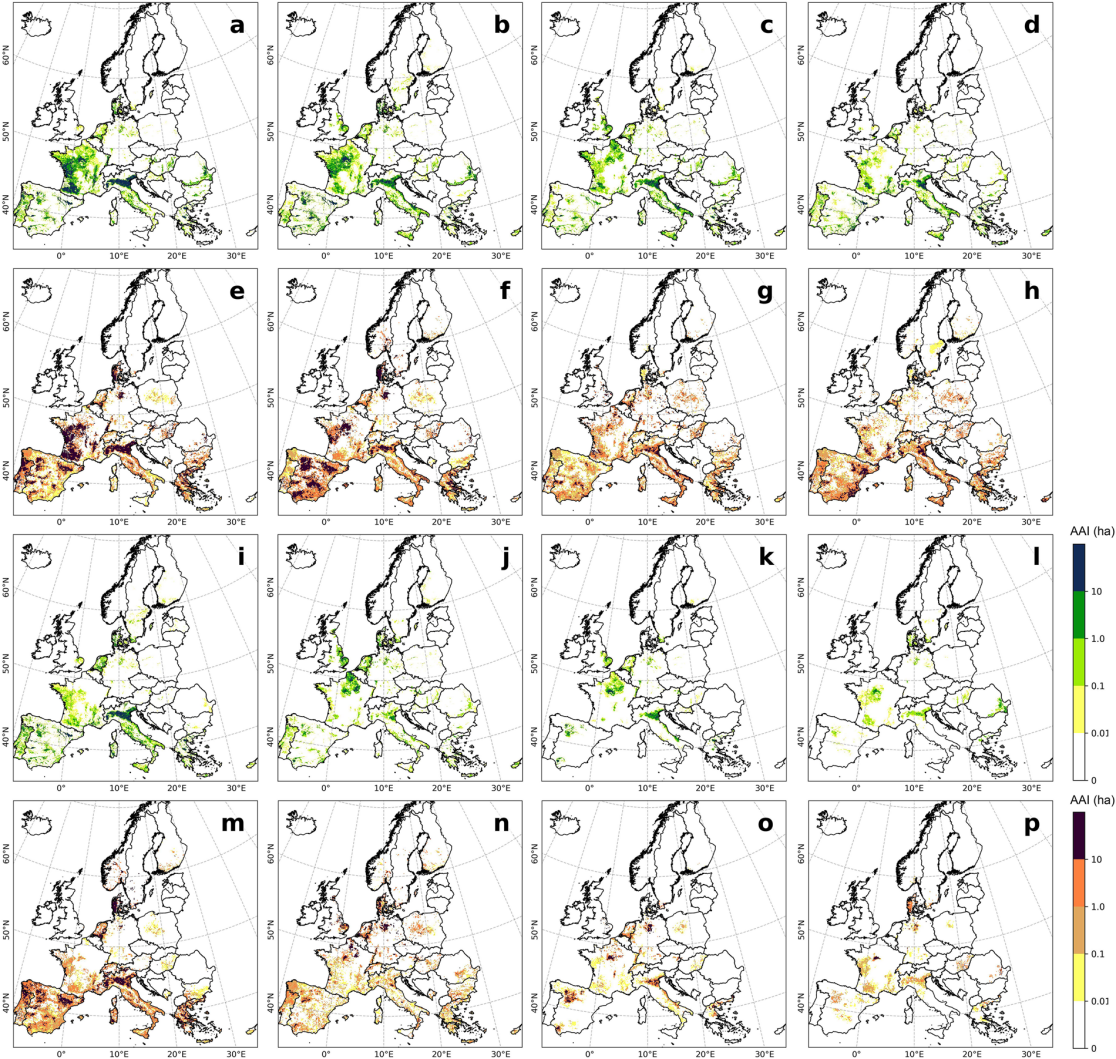
Crop-specific irrigated area in ECIRA and EIM datasets in 2010. ECIRA data: (**a**) maize, (**b**) other cereals (excluding maize and rice), (**c**) fresh vegetable, melon, and strawberry in open fields, (**d**) fruit and berry, (**i**) grassland, (**j**) potato, (**k**) sugar beet, and (**l**) rapeseed. EIM 2010 data: (**e**) maize, (**f**) other cereals (excluding maize and rice), (**g**) fresh vegetable, melon, and strawberry in open fields, (**h**) fruit and berry, (**m**) grassland, (**n**) potato, (**o**) sugar beet, and (**p**) rapeseed. Specific crop type classification is shown in Table 4.

**Fig. 10:**
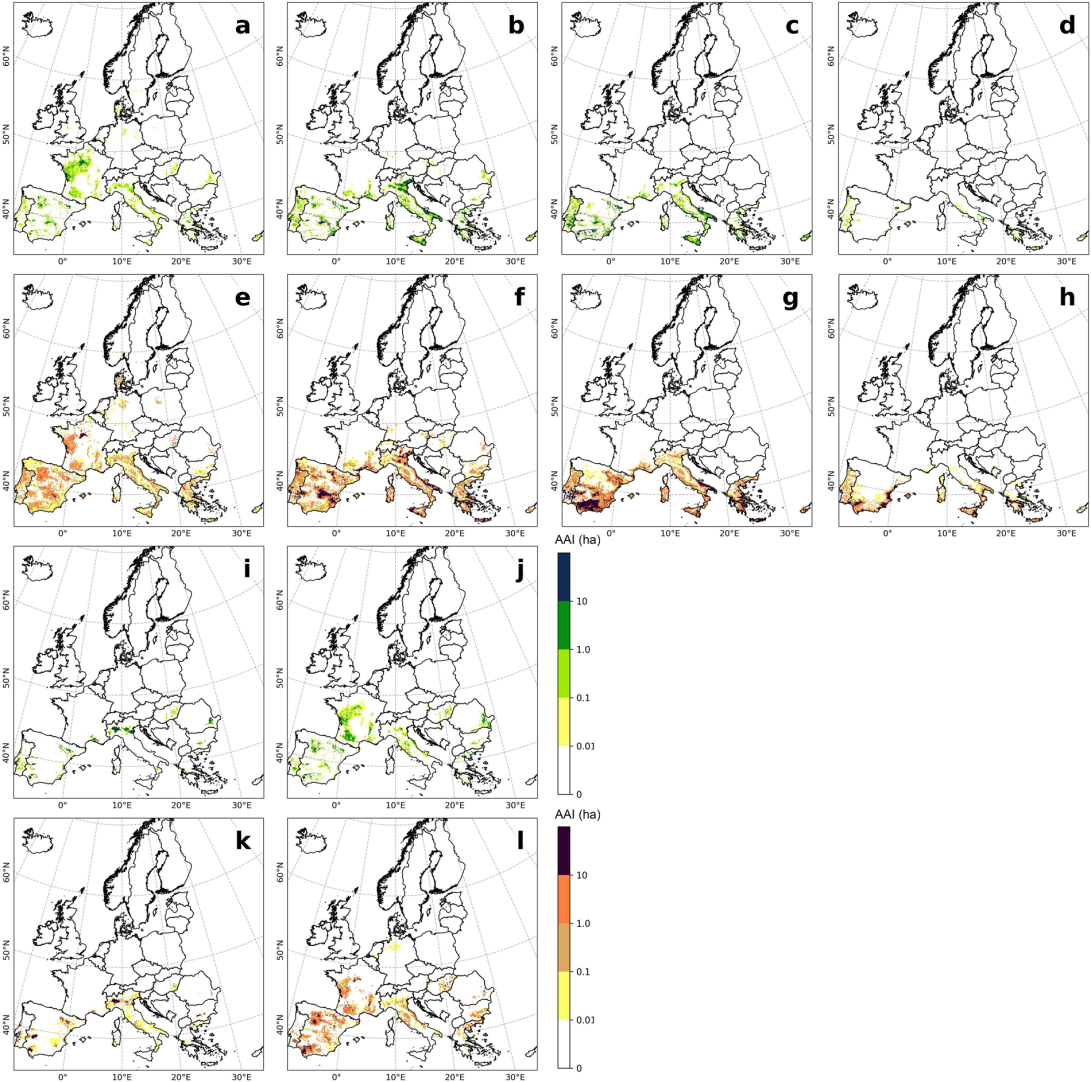
Crop-specific irrigation area in ECIRA and EIM datasets in 2010. ECIRA data: (**a**) pulses, (**b**) vineyard, (**c**) olive, (**d**) citrus, (**i**) rice, and (**j**) sunflower. EIM 2010 data: (**e**) pulses, (**f**) vineyard, (**g**) olive, (**h**) citrus, (**k**) rice, and (**l**) sunflower. Specific crop type classification is shown in Table 4.

**Fig. 11:**
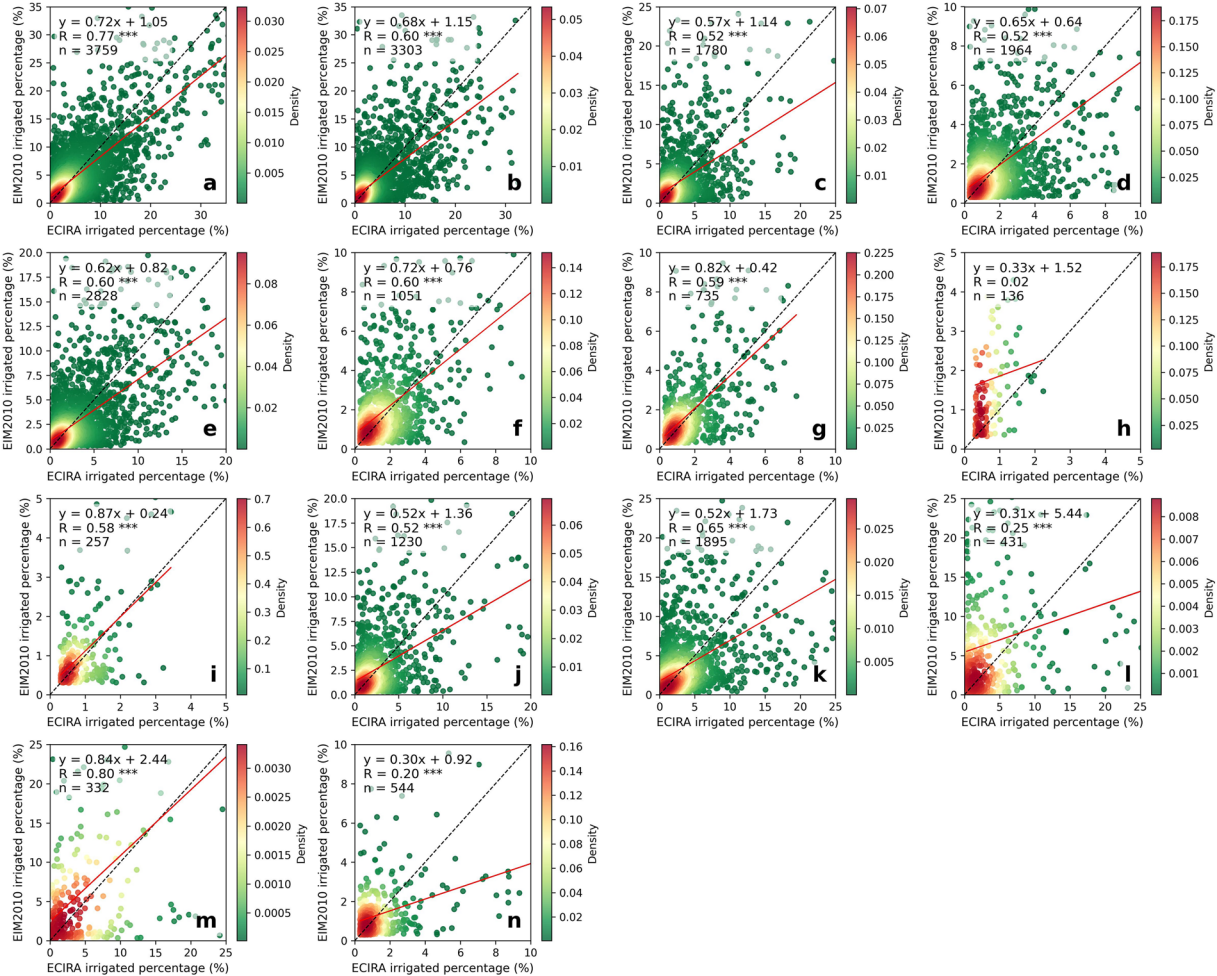
Comparison of ECIRA and EIM 2010 irrigated percentages at 10 km grid level. (**a**) Maize, (**b**) other cereals (excluding maize and rice), (**c**) fresh vegetable, melon, and strawberry in open fields, (**d**) fruit and berry, (**e**) grassland, (**f**) potato, (**g**) sugar beet, (**h**) rapeseed, (**i**) pulses, (**j**) vineyard, (**k**) olive, (**l**) citrus, (**m**) rice, and (**n**) sunflower. Grids with irrigated percentages lower than 0.30% were not included in this comparison.

The comparison between ECIRA and EIM 2010 across 14 crop types (Table 4) revealed that, in southern EU regions, EIM 2010 identified a broader distribution and greater extent of irrigated areas compared to ECIRA, particularly in the Iberian Peninsula and the southern Balkan Peninsula (especially Greece) (Figs. 9, 10). In contrast, in western EU regions, ECIRA reported more extensive irrigated areas in northern France and eastern Great Britain, especially for other cereals, potatoes, and sugar beet. In central and eastern EU regions, EIM 2010 detected a larger crop-specific irrigated area in Bulgaria, whereas ECIRA identified a greater irrigated area in eastern Romania. In northern EU regions, both datasets captured the limited crop-specific irrigated extent and exhibited good spatial consistency. Overall, while ECIRA and EIM 2010 both effectively depicted crop-specific irrigation hotspots, EIM 2010 revealed a wider distribution of irrigated areas in these hotspot regions, whereas ECIRA identified a more extensive range of crop-specific irrigated areas in relatively humid and temperate regions of Europe. For the grid-wise comparison (Fig. 11), only rapeseed, citrus, and sunflower showed weak agreement, while other crop types exhibited moderate to strong agreement, with R values ranging from 0.52 to 0.80.

The primary data source for both ECIRA and EIM 2010 is the Eurostat farm structure survey, which underpins their overall alignment. However, the coarser spatial resolution of EIM 2010 (10 km) compared to ECIRA (1 km) may lead to the omission of smaller irrigated areas and inaccuracies in representing irrigation patterns, particularly in regions with fragmented irrigated farmland and less frequent irrigation. Moreover, variations in disaggregation strategies and spatial resolutions of the data sources further contribute to the discrepancies between ECIRA and EIM 2010. Specifically, EIM 2010 was generated using 10-km gridded total AEI, total AAI, and total crop growing area without crop type classification, along with subnational crop-specific AAI. In contrast, ECIRA was developed using total AEI at 5 arc-minute resolution, subnational total AAI, 1-km gridded crop-specific growing areas, and subnational crop-specific AAI. EIM 2010 established irrigation priorities for disaggregating total gridded AAI to various crops based on factors such as mandatory irrigation needs, crop economic value, and the ratio of irrigated to total crop-growing area within a region^3^. The disaggregated crop-specific AAI was constrained by gridded total crop and irrigated areas, as well as subnational crop-specific irrigated areas. As a result, EIM 2010 might omit irrigated area for crops with lower irrigation priority (such as cereals and grassland) or small cultivation areas within a region. In contrast, ECIRA does not incorporate irrigation priorities and instead focuses on the spatial patterns of irrigation infrastructure and crop-specific growing areas. The disaggregated 1-km gridded crop-specific AAI in ECIRA is constrained by crop-specific NUTS2-level totals, enabling more accurate detection of irrigation even for crops with smaller irrigated areas.

## Usage Notes

This study produced annual European Crop-specific IRrigated Area (ECIRA) data at a 1 km × 1 km resolution with an EPSG 3035 projection for the years 2010–2020, covering 28 European countries using a simple yet effective method. Comparisons with other statistical survey-based datasets demonstrated that ECIRA effectively identified crop-specific irrigated hotspots and exhibited great potential for revealing the interannual dynamic spatial patterns of irrigation, particularly in the temperate zones of Europe. To demonstrate interannual variability, we compared irrigated areas in 2017 (a relatively wet year) and 2018 (a dry year) in Northwestern Europe. Potato and sugar beet were selected as representative crops due to their regional importance and sensitivity to water availability. As shown in Fig. S7, the irrigated area expanded notably in 2018 compared to 2017, suggesting that ECIRA has the potential to reflect year-to-year irrigation dynamics.

Due to current data limitations, it is not feasible to directly evaluate ECIRA at the 1 km scale. Nevertheless, comparisons with coarser-resolution datasets (around 10 km) can still indirectly demonstrate the reliability of ECIRA in capturing the spatial distribution and interannual dynamics of crop-specific irrigation. In addition, the consistency between ECIRA and point-based *in-situ* survey data from LUCAS (conducted at 2 km grid) also provides supporting evidence. These validation efforts represent the most robust assessments that can currently be conducted given the available data. This survey data-based ECIRA dataset addresses limitations faced by remote sensing and is therefore anticipated to outperform remote sensing in detecting irrigation areas in humid regions of Europe. Besides, high-spatial resolution crop-specific irrigated area datasets are available for certain countries, such as Italy and Spain, where irrigation is essential for sustaining agricultural production. For studies targeting specific countries or local regions, these region-specific datasets are generally more appropriate than broader European-scale datasets like ECIRA, as they typically offer higher accuracy and are better tailored to the unique characteristics of the local agricultural systems.

Crop classification in ECIRA primarily follows the Eurostat standard which specifies 46 crop types from the European Agriculture Census 2010. However, due to data limitations, these crop types are aggregated into 16 categories within ECIRA. Notably, the Grassland category in ECIRA includes fallow land, as this classification is determined by the DGPCM dataset. The ‘other crops’ category aggregates many crop types and covers a large irrigated area, making it challenging to simulate crop-specific water use due to the difficulty in determining parameters for the diverse crop types included. We maintain consistency between the crop-specific irrigated area sum in ECIRA and the total irrigated area in ELIAD, because the total irrigated area in ELIAD is not only dynamic annually but also estimated by considering appropriate irrigation reference years, the availability of irrigation infrastructure, and the impact of climatic conditions on the use of irrigation infrastructure – factors not considered in the Eurostat 2010 dataset. Consequently, the sum of crop-specific irrigated areas in ECIRA differs from that in Eurostat 2010. Notably, the Eurostat 2010 dataset does not always maintain consistency between the total irrigated area and the sum of crop-specific irrigated areas, but we did not adjust these indicators due to a lack of additional information. To ensure that the sum of crop-specific irrigation proportions equals 100%, we used the crop-specific irrigated area sum rather than the reported total irrigated area from Eurostat 2010 for disaggregating ELIAD total irrigated area to different crop types.

The high spatiotemporal resolution of ECIRA is achieved through necessary assumptions due to limited data availability and the complexity of real conditions. As these introduce uncertainties and limitations, there is still potential for improvements. Firstly, while the development of the annual total AAI from the ELIAD dataset—the primary input for ECIRA—already accounted for the annual temporal dynamics of irrigation equipment at the NUTS2 level, the 1-km gridded irrigation equipment conditions for ECIRA development were kept constant, relying on 0.6-arc minute AEI data from 2005 and 5-arc minute AEI data from 2010. It has been shown that the total irrigable area in Southern EU regions, where irrigation is critical for agricultural productivity, has remained relatively stable during this period, while it has increased in temperate regions like parts of Western and Central Europe and decreased in parts of Northern Europe^26^. When gridded irrigable area data for 2020 becomes available, simple interpolation can be applied to fill the temporal gaps for the 2010–2020 period, enabling a more accurate characterization of year-specific spatial patterns in areas with irrigation infrastructure. Additionally, generating the total irrigable area data with a higher spatial resolution in 2010 involves integrating corresponding data from different scales and years: 2010 (low spatial resolution, 5 arc minutes) and 2005 (high spatial resolution, 0.6 arc minutes). This process of irrigable area data resampling and integration may introduce new uncertainties.

Secondly, our method for disaggregating the total irrigated area to different crops assumed uniform irrigation percentages (i.e., the ratio of irrigated area to irrigable area) across all 1 km grids within a specific NUTS2 region, which did not account for the spatial heterogeneity of irrigation percentages. Furthermore, subnational crop-specific irrigated area data is only available for the reference year 2010 within the 2010–2020 period. As a result, we assumed that the proportion of crop-specific irrigated area relative to the total irrigated area remained constant throughout this decade. However, the crop irrigation proportion and the spatial patterns of irrigation percentage are likely to fluctuate over time due to factors such as economic and policy factors, freshwater availability, and climate variability^57–59^. Nevertheless, since we provide the Python codes and detailed methodology for generating ECIRA, future updates and improvements to the ECIRA data can be carried out using the approach outlined in this study once more recent irrigation data becomes available. Finally, there are inherent limitations with survey data, such as variability in data collection methodologies across countries and potential systematic reporting biases^60,61^. However, integrating high-temporal and spatial resolution remote sensing data with survey data could address these issues and significantly enhance the reliability and accuracy of crop-specific irrigated area datasets.

Crop-specific annual irrigated area is presented in GeoTiff format, each GeoTiff shows the irrigated area of a crop in a year. These raster datasets can be easily imported into ArcGIS and ENVI for further analyses.

## Supplementary information

Supplementary information

## Data Availability

In this study, Python 3.10 is used to generate the ECIRA dataset. The corresponding python scripts are available at: https://github.com/Wanxuezhu666/ECIRAv2-processing-details.git.
