# Supplementary material for: ECIRA - European crop-specific irrigated area at 1 km resolution annually from 2010 to 2020
Source: Sci Data. 2025 Aug 4;12:1349. doi: 10.1038/s41597-025-05628-y (PMC12322114; doi:10.1038/s41597-025-05628-y)
Supplement: Supplementary file 1 — Supplementary information [file 41597_2025_5628_MOESM1_ESM.pdf]

Supplementary information

ECIRA: Annual 1 km crop-specific irrigated and rainfed area across Europe from 2010 to 2020 (Version 2.0)

Wanxue Zhu[1, *], Josef Baumert[2], Hugo Storm[2], Thomas Heckelei[2], Stefan Siebert[1]

1. Department of Crop Sciences, University of Göttingen, Von-Siebold-Str. 8, 37075 Göttingen, Germany
2. Institute for Food and Resource Economics (ILR), University of Bonn, Germany
* Corresponding author: wanxue.zhu@agr.uni-goettingen.de

Updated: 05 June, 2025

Introduction

The **European Crop-specific IRrigated Area Dataset (ECIRA)** provides data on the irrigated and rainfed area for specific crops across 28 European countries from 2010 to 2020, with a spatial resolution of 1 km and using the EPSG: 3035 projection. ECIRA includes 16 crop types: cereals (excluding maize and rice), maize, rice, pulses, potatoes, sugar beets, rapeseed and turnip rapeseed, sunflower, textile crops, open-field vegetables, melons, strawberries, grasslands, fruits and berries, citrus, olives, vineyards, and other crops.

The 28 European countries include Austria, Belgium, Bulgaria, Cyprus, the Czech Republic, Germany, Denmark, Estonia, Greece, Spain, Finland, France, Croatia, Hungary, Ireland, Italy, Lithuania, Luxembourg, Latvia, Malta, Netherlands, Poland, Portugal, Romania, Sweden, Slovenia, Slovakia, and the United Kingdom

This document provides the supplementary tables and figures referenced in the manuscript.

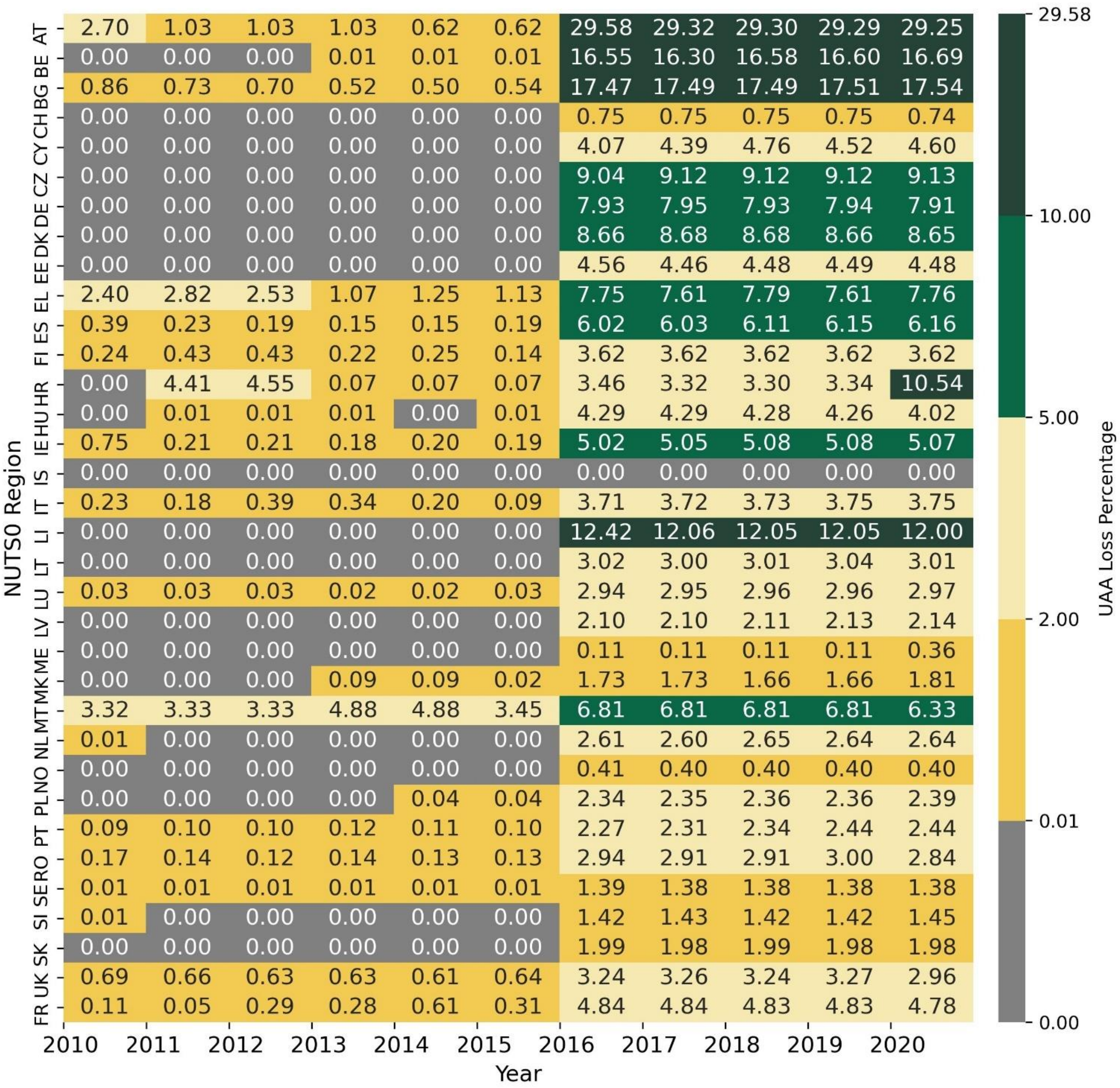

Figure S1. Percentage of UAA loss to total UAA (%) at the country level from the year 2010 to 2020. Abbreviations of NUTS0 are shown in Table S1.

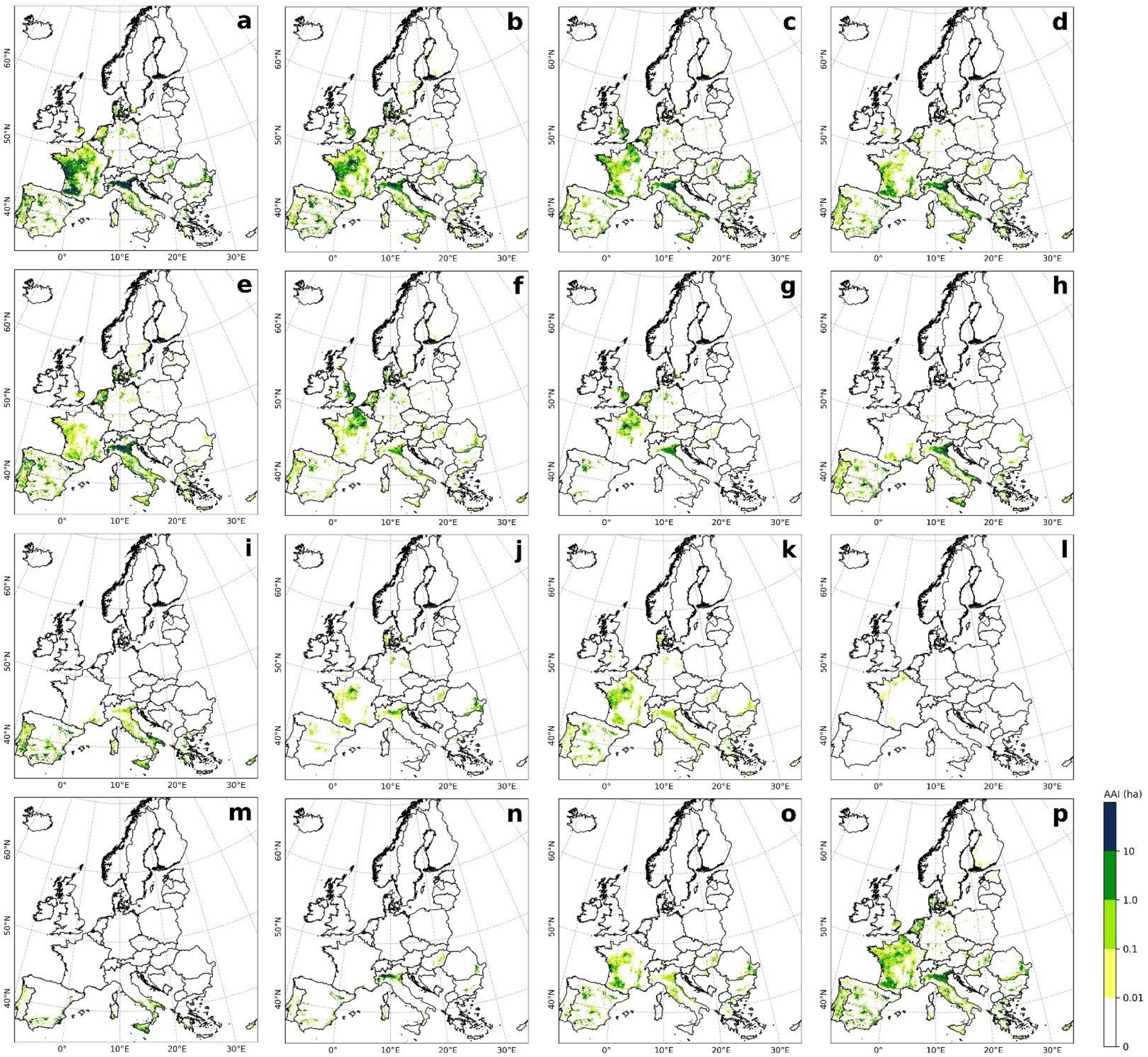

Figure S2. Crop-specific irrigated area in the ECIRA dataset in 2012.

(**a**) Maize, (**b**) other cereals (excluding maize and rice), (**c**) vegetables, strawberry, and melon in open field, (**d**) fruit and berry, (**e**) grassland, (**f**) potato, (**g**) sugar beet, (**h**) vineyard, (**i**) olive, (**j**) rape and turnip rape, (**k**) pulses, (**l**) textile crops, (**m**) citrus, (**n**) rice, (**o**) sunflower, and (**p**) other crops. Crop type classification is shown in Table 3 of the main manuscript.

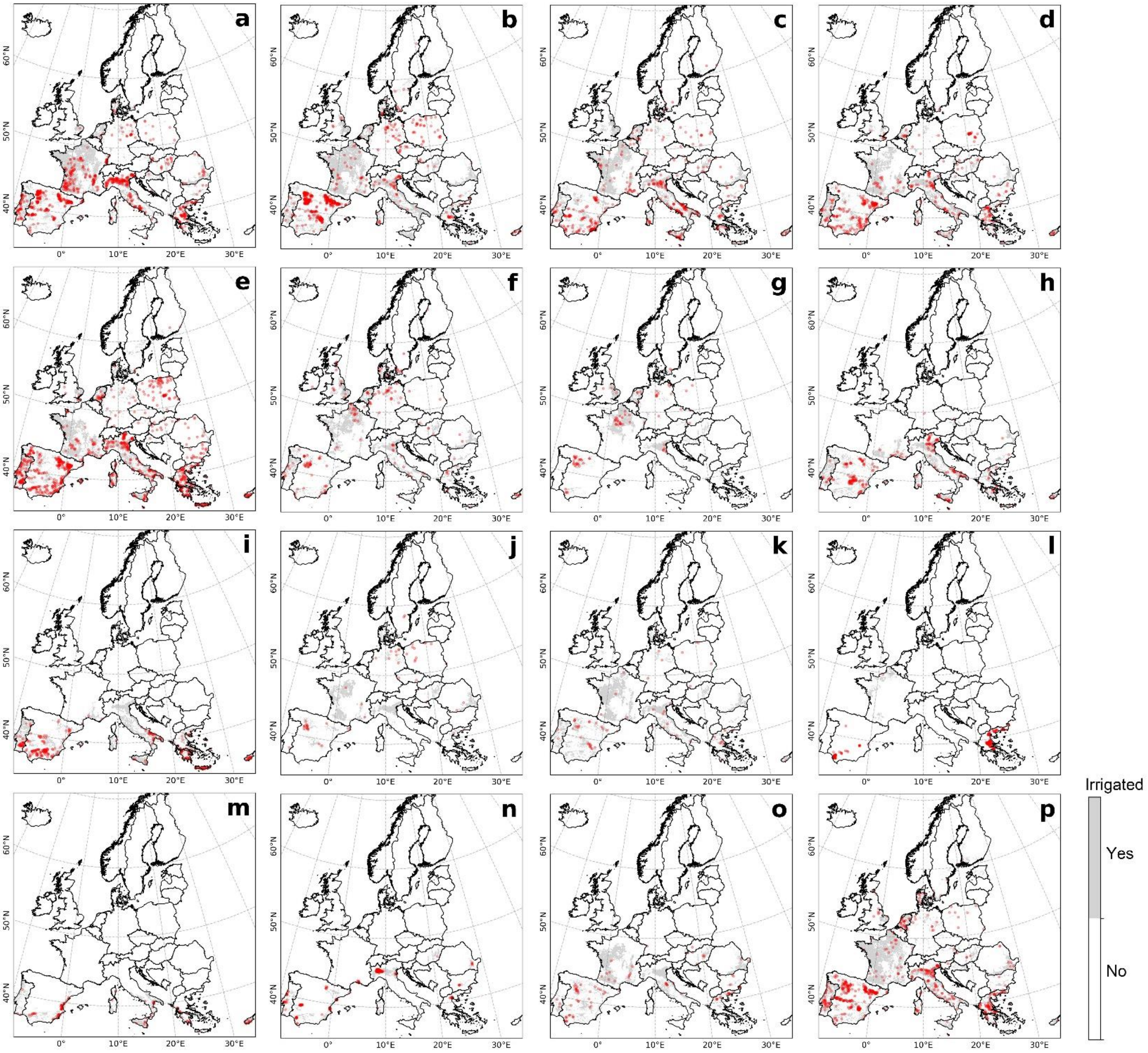

Figure S3. Crop-specific irrigated points in the LUCAS dataset in 2012.

(**a**) Maize, (**b**) other cereals (excluding maize and rice), (**c**) fresh vegetables, strawberry, and melon in open field, (**d**) fruit and berry, (**e**) grassland, (**f**) potato, (**g**) sugar beet, (**h**) vineyard, (**i**) olive, (**j**) rape and turnip rape, (**k**) pulses, (**l**) textile crops, (**m**) citrus, (**n**) rice, (**o**) sunflower, and (**p**) other crops. The red points are irrigated points with LUCAS water management labels of 'irrigation' and 'potential irrigation'; the light grey background is the irrigated area (> 0 ha) identified in ECIRA. Crop type classification is shown in Table 4 of the main manuscript.

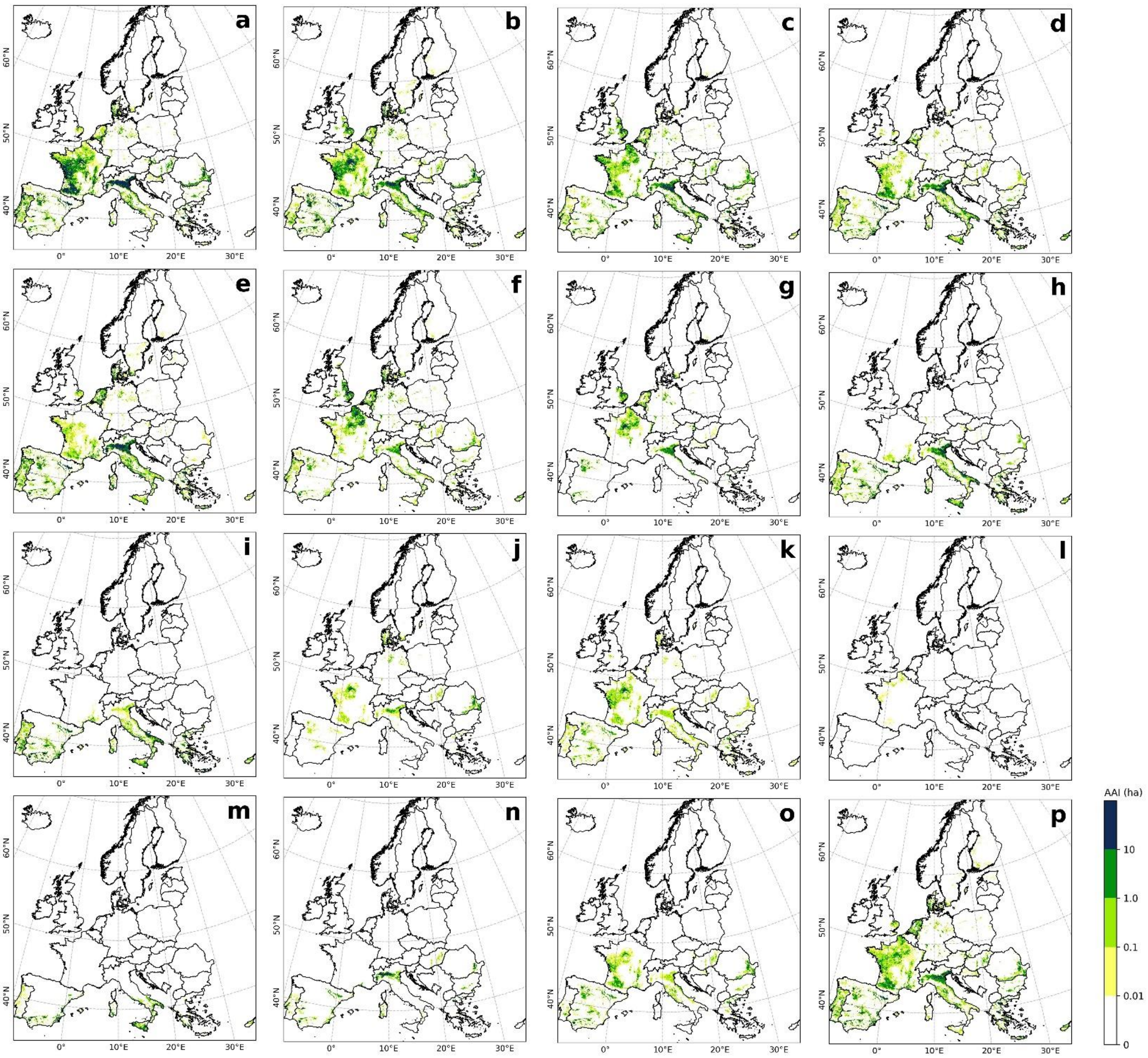

Figure S4. Crop-specific irrigated area in the ECIRA dataset in 2015.

(**a**) Maize, (**b**) other cereals (excluding maize and rice), (**c**) fresh vegetables, strawberry, and melon in open field, (**d**) fruit and berry, (**e**) grassland, (**f**) potato, (**g**) sugar beet, (**h**) vineyard, (**i**) olive, (**j**) rape and turnip rape, (**k**) pulses, (**l**) textile crops, (**m**) citrus, (**n**) rice, (**o**) sunflower, and (**p**) other crops.

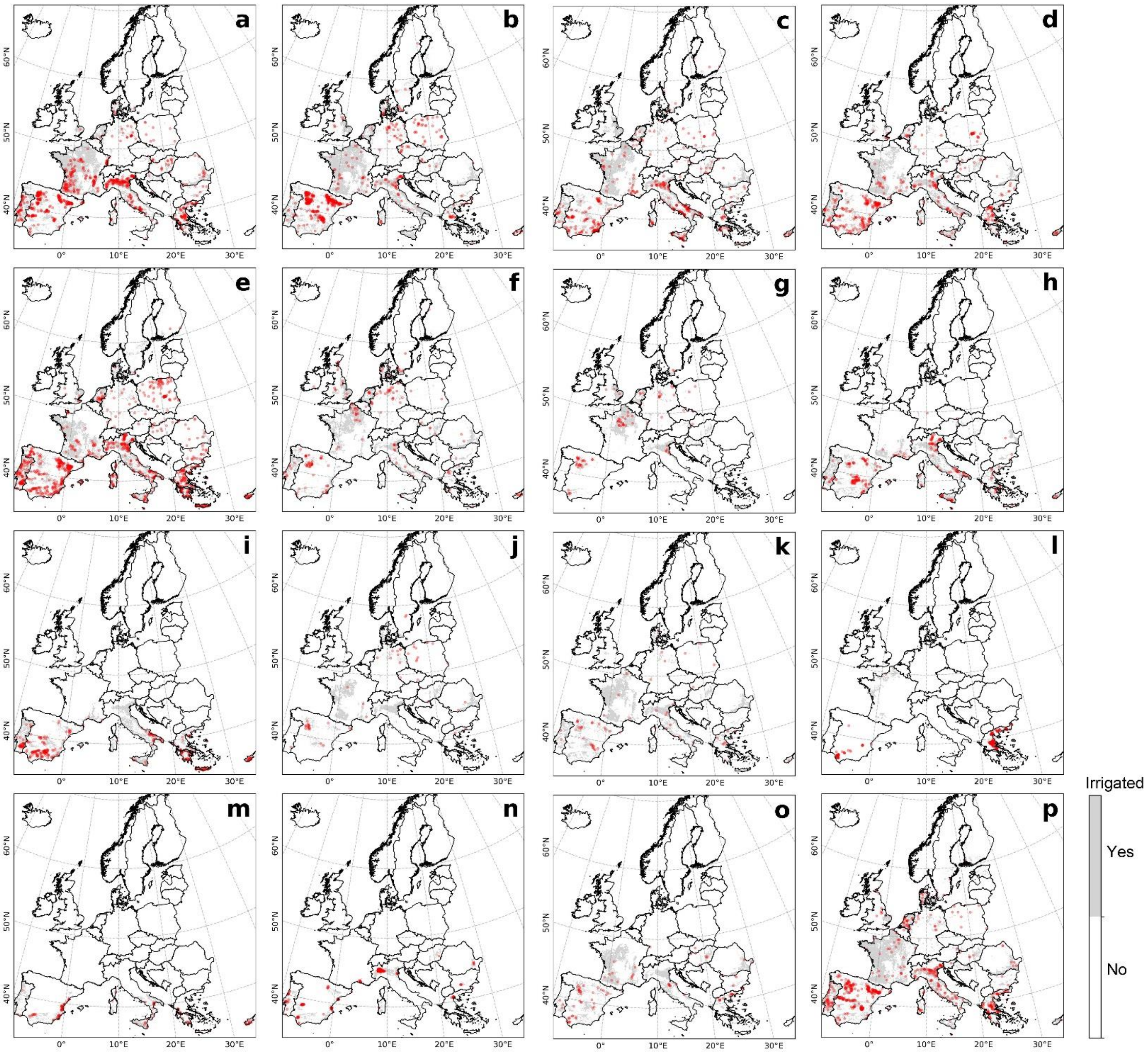

Figure S5. Crop-specific irrigated points in the LUCAS dataset in 2015.

(**a**) Maize, (**b**) other cereals (excluding maize and rice), (**c**) fresh vegetables, strawberry, and melon in open field, (**d**) fruit and berry, (**e**) grassland, (**f**) potato, (**g**) sugar beet, (**h**) vineyard, (**i**) olive, (**j**) rape and turnip rape, (**k**) pulses, (**l**) textile crops, (**m**) citrus, (**n**) rice, (**o**) sunflower, and (**p**) other crops. The red points are irrigated points with LUCAS water management labels of 'irrigation' and 'potential irrigation'; the light grey background is the irrigated area (> 0 ha) identified in ECIRA.

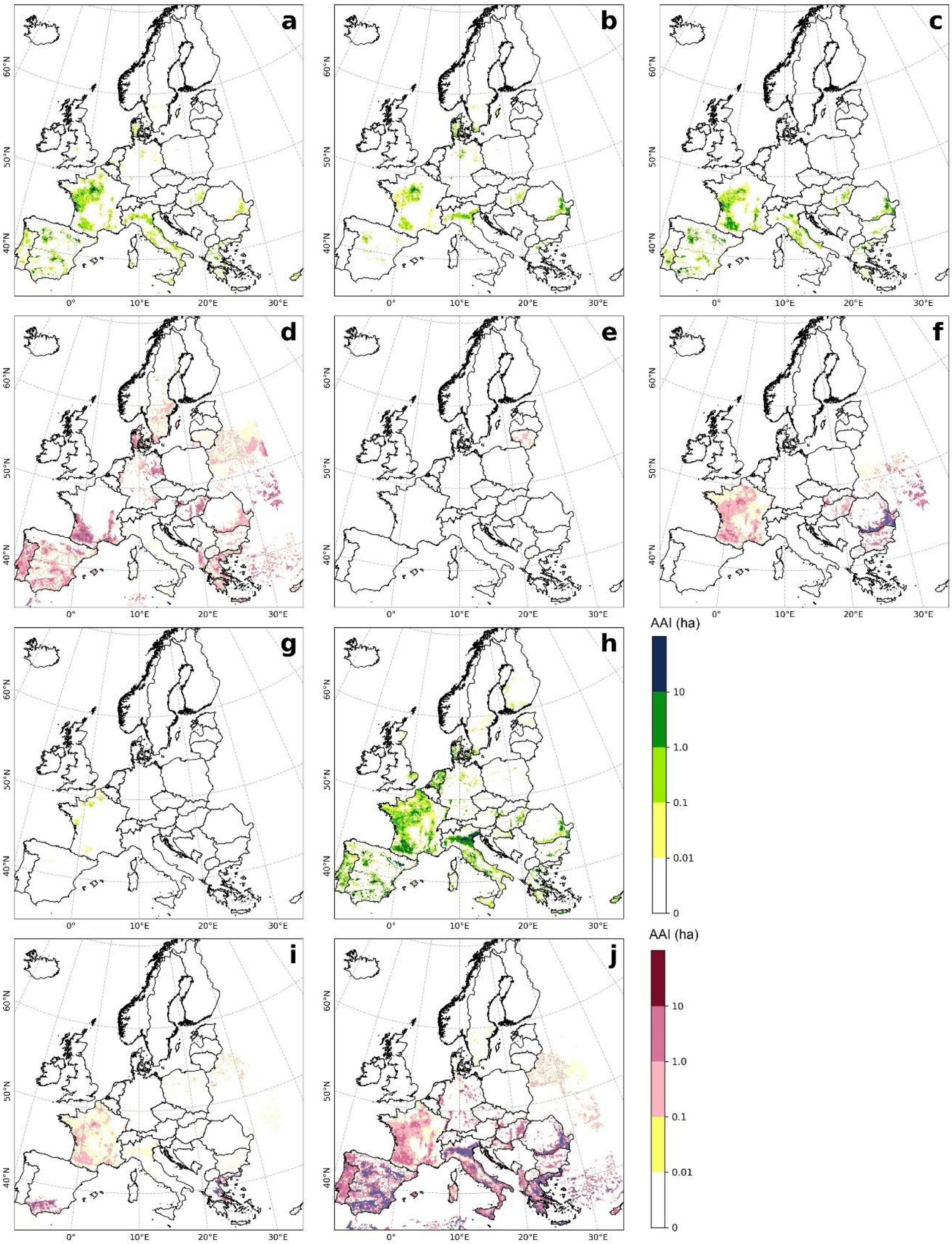

Figure S6. Crop-specific irrigation area in ECIRA and SPAM datasets in 2010. AAI data for ECIRA dataset: (**a**) pulses, (**b**) rape, (**c**) sunflower, (**g**) textile crops, and (**h**) other crops. AAI data for SPAM dataset: (**d**) pulses, (**e**) rape, (**f**) sunflower, (**i**) textile crops, and (**j**) other crops. Specific crop type classification is shown in Table 4 of the main manuscript.

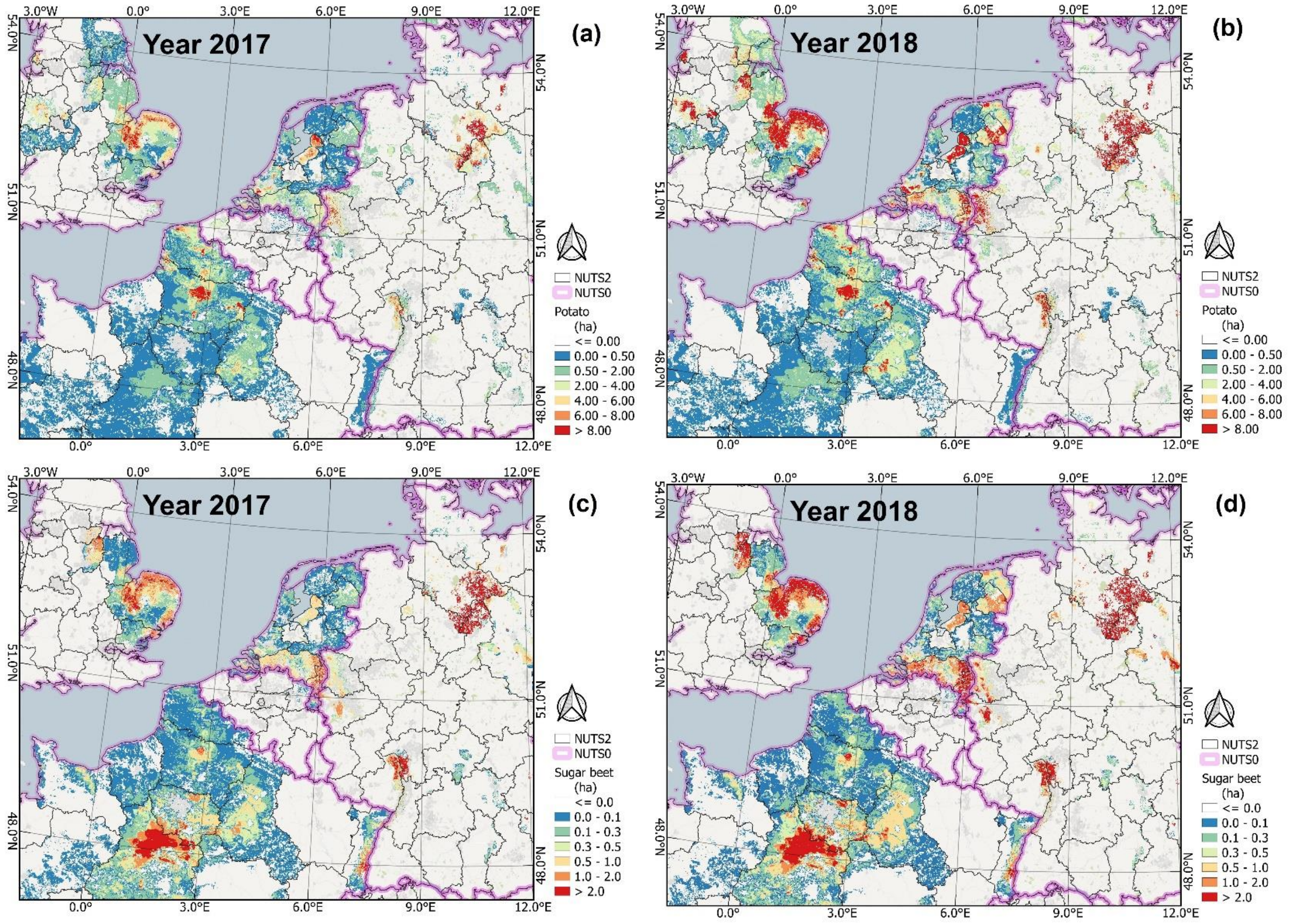

Figure S7. Comparison of irrigated area in Northwestern Europe in 2017 (wet year) and 2018 (dry year). Irrigated area (AAI) of potato in 2017 (**a**) and 2018 (**b**); irrigated area of sugar beet in 2017 (**c**) and 2018 (**d**).

Table S1 Twenty-eight EU countries (NUTS0 code) selected for developing ECIRA

Southern EU	Central EU	Eastern EU	Western EU	Northern EU
Cyprus (CY)	Austria (AT)	Bulgaria (BG)	Belgium (BE)	Denmark (DK)
Greece (EL)	Czech Republic (CZ)	Estonia (EE)	Luxemburg (LU)	Finland (FI)
Spain (ES)	Germany (DE)	Lithuania (LT)	Netherlands (NL)	Sweden (SE)
France (FR)	Croatia (HR)	Latvia (LV)	United Kingdom (UK)	Ireland (IE)
Italy (IT)	Hungary (HU)	Poland (PL)		
Malta (MT)	Slovenia (SI)	Romania (RO)		
Portugal (PT)	Slovakia (SK)			

Note: Northern Europe experiences a cold climate with no dry season but relatively warm summers, gradually transitioning to a milder, temperate climate as one moves toward central and western Europe. Heading further south in Europe, a temperate climate with dry summers and partly dry winters prevails with a transition to semi-arid conditions in southern Spain. France is assigned to Southern Europe, due to the substantial irrigation extent in southern France.

Table S2 Comparison of irrigated area in selected Northwest European countries between 2017 (wet year) and 2018 (dry year)

	Total (10^3 ha)		Potato (10^3 ha)		Sugar beet (10^3 ha)	
NUTS0	2017	2018	2017	2018	2017	2018
FR	1678	1784	87	111	38	49
DE	314	611	73	145	27	53
NL	162	377	28	69	6.2	15
UK	135	300	59	137	11	23
DK	179	244	16	21	-	-
BE	102	190	1.3	2.3	0.075	0.143
